# Integrating ferroptosis and the tumor-immune gene mutations interface: a novel lenvatinib resistance prognostic signature with YWHAB-related immunotherapeutic correlates in hepatocellular carcinoma

**DOI:** 10.3389/fimmu.2026.1913042

**Published:** 2026-10-29

**Authors:** Hang Yu, Zhihui Wang, Hongliang Zou, Haikuan Liu, Yujie Ma, Baoshan Cao, Jie Wen, Abudureyimujiang Aili

**Affiliations:** 1 Department of Interventional Oncology, The First Affiliated Hospital of Sun Yat-sen University, Sun Yat-sen University, Guangzhou, China; 2 Department of Medical Oncology and Radiation Sickness, Peking University Third Hospital, Beijing, China

**Keywords:** ferroptosis, HCC, lenvatinib resistance, PI3K/AKT, YWHAB

## Abstract

**Background:**

Lenvatinib resistance represents a major clinical challenge in the treatment of hepatocellular carcinoma (HCC), necessitating the identification of strategies to overcome this issue. Although ferroptosis, a non-apoptotic cell death process, is implicated in the progression of HCC and the response to lenvatinib, the prognostic relevance and the lenvatinib response-related mechanisms of ferroptosis-related genes (FRGs) remain poorly understood.

**Methods:**

An eight-gene ferroptotic lenvatinib-related prognostic signature (FLRPS) was developed as a prognostic model. Based on the median risk score, the patients were classified into high-risk and low-risk groups to compare clinical outcomes, immune traits, and mutational profiles. Immunohistochemistry, spatial transcriptome analysis and single-cell RNA sequencing (scRNA-seq) were used to identify the specificity of YWHAB expression in HCC. *In vitro* and *in vivo* assays were utilized to evaluate the impact of YWHAB on the phenotypes of HCC cells and lenvatinib-induced ferroptosis, as well as to investigate the underlying mechanisms.

**Results:**

The FLRPS, comprising eight genes (CENPA, RAN, CDCA8, KIF2C, YWHAB, HDAC2, DYNC1H1, and NRAS), yielded scores that positively correlated with poorer prognosis, advanced pathological grade, later clinical stage, metrics of genomic instability, and lower M2 macrophage infiltration. Furthermore, a nomogram integrating the FLRPS and tumor stage accurately predicted overall survival. Additionally, lenvatinib-resistant HCC samples exhibited elevated YWHAB expression. Downregulation of YWHAB sensitized Hepa1–6 cells to lenvatinib both *in vitro* and *in vivo*, an effect accompanied by decreased GPX4 levels and reversed by liproxstatin-1, suggesting a ferroptosis-related mechanism involving PI3K/AKT pathway inhibition.

**Conclusions:**

The FLRPS serves as an effective predictive model for the prognosis of HCC. Targeting YWHAB may represent a promising therapeutic strategy to overcome lenvatinib resistance in HCC.

## Introduction

Hepatocellular carcinoma (HCC) is the third leading cause of cancer-related death worldwide, presenting a rising global incidence (1) and approximately 865,269 new cases reported annually (2). The pathogenesis of HCC involves multiple etiologies, including non-alcoholic fatty liver disease, cirrhosis, and viral hepatitis (3). Owing to the late onset of symptoms, up to 50% of patients are diagnosed at an advanced stage (4). Despite advances in immunotherapy and targeted therapy (5), the 5-year survival rate remains below 15% (6). Lenvatinib, a first-line multi-targeted tyrosine kinase inhibitor, demonstrates clinical efficacy by targeting VEGFR, FGFR, PDGFR, KIT, and RET (7). However, the effectiveness of lenvatinib is limited by acquired drug resistance, and the mechanisms underlying this resistance remain largely unknown.

Ferroptosis is a distinct form of programmed cell death driven by the accumulation of reactive oxygen species (ROS) and intracellular iron, along with peroxidation of unsaturated fatty acids (PUFAs) (8), representing a promising therapeutic strategy in cancer (9). This process is primarily regulated through the inhibition of GPX4, which disrupts glutathione-dependent antioxidant defenses and leads to uncontrolled lipid peroxidation and membrane damage (10). Recently, lenvatinib has been shown to trigger ferroptosis in HCC through multiple reported mechanisms (11–20). Collectively, this evidence highlights the crucial role of ferroptosis in modulating the response to lenvatinib in HCC, which may be attributed to the iron-rich hepatic milieu and the central involvement of the liver in the metabolism of ROS and lipids (including PUFAs) (21–23). Nevertheless, the association between ferroptosis-related genes (FRGs) and lenvatinib resistance in HCC has not been systematically elucidated, and the functions of numerous lenvatinib-associated ferroptotic genes in HCC remain largely unexplored.

In this study, we aimed to construct and validate a ferroptosis- and lenvatinib-resistance-associated prognostic signature for HCC, and investigate whether YWHAB, a candidate signature gene, modulates lenvatinib sensitivity through ferroptosis and the PI3K/AKT pathway.

## Materials and methods

### Statistical analysis

All statistical analyses in this study were conducted using R software (version 4.2.2) and GraphPad Prism 8. Survival curves were generated using the Kaplan-Meier (K-M) method via the survival (version 3.3.1) and survminer (version 0.4.9) R packages, and the statistical significance of the differences was assessed using the log-rank test. The predictive accuracy of the FLRPS and the integrated nomogram was further evaluated via time-dependent receiver operating characteristic (ROC) curve analysis. The unpaired, two-tailed Student t-test was utilized for normally distributed data, whereas the Mann-Whitney U test was applied for non-parametric comparisons. A P-value of< 0.05 was considered to indicate statistical significance across all tests. The results are denoted as follows: * P< 0.05, ** P< 0.01, *** P< 0.001, and **** P< 0.0001. Two-tailed Student’s t-tests (paired and unpaired) were performed on normally distributed data. The number of biological replicates and the definition of error bars (mean ± SEM) are stated in the corresponding figure legends.

### Data collection

Ethics committee approval was not required for the analyses of public databases. The RNA-sequencing (RNA-seq) data, alongside the corresponding clinical information and single nucleotide variant (SNV) data for LIHC, were downloaded from the TCGA dataset (https://xenabrowser.net/datapages/) (369 HCC samples and 50 normal samples). Clinical characteristics were acquired via the “cgdsr” R package, and mutation data were obtained using the “TCGAbiolinks” R package. Furthermore, the RNA-seq data from 232 patients with HCC were downloaded from the International Cancer Genome Consortium (ICGC) portal (https://dcc.icgc.org/projects/LIRI-JP/) to serve as the validation cohort. Data regarding the immunotherapy cohorts were retrieved from the IMvigor210 database (http://research-pub.Gene.com/IMvigor210CoreBiologies) (348 samples). Based on data from FerrDb database (http://www.zhounan.org/ferrdb/) and previous literature (24, 25), 291 FRGs were obtained and designated as FRGs-set1 (**Supplementary Table 1**). To comprehensively identify the lenvatinib resistance associated genes set (LRAGS), data from three GEO datasets (GSE273819 (26), GSE186191 (27), and GSE252752 (28)), were integrated and analyzed. The GSE273819 dataset captured transcriptomic alterations in an established lenvatinib-resistant PLC/PRF/5 HCC cell line. The GSE186191 dataset provided paired transcriptional profiles of parental and lenvatinib-resistant Hep3B and Huh7 cells. The GSE252752 dataset contained RNA-seq data from orthotopic xenograft HCC tumors (MHCC97H). For LRAGS construction, only the untreated control group (GSM8006337, GSM8006338, and GSM8226341) and the lenvatinib-treated group (GSM8006343, GSM8006344, and GSM8006345) were used. Differentially expressed genes (DEGs) between comparative subgroups in each dataset were identified using the limma package in R, with a significance cutoff set at |log_2_fold change (FC)| > 0.585 and adjusted *P-value< 0.05*. To maximize coverage of candidate genes involved in lenvatinib resistance, the DEGs from these three datasets were combined into a union set of 6,818 protein-coding genes, which was designated as the LRAGS. Additional public datasets downloaded from the GEO database were used for validation and treatment-response analyses, including GSE14520 (221 HCC patients with survival data), GSE76427 (an HCC cohort), and GSE109211 (sorafenib-treated HCC samples).

### Cell culture

The mouse HCC cell line Hepa1–6 was obtained from the American Type Culture Collection (ATCC). Cells were cultured in Dulbecco’s Modified Eagle Medium (DMEM) supplemented with 10% fetal bovine serum (FBS) and 1% penicillin/streptomycin at 37 °C in a humidified atmosphere containing 5% CO_2_.

### Enrichment analysis

Based on gene expression profiles from the TCGA-HCC cohort, DEGs between tumor tissues and adjacent normal controls were identified using the “limma” R package (v4.1.3) (29) with cutoff criteria set at |log_2_FC| > 1 and adjusted *p< 0.05*. Subsequently, KEGG pathway enrichment analysis of the identified DEGs was performed using the Metascape online platform (https://metascape.org) (30). Genes associated with the ferroptosis pathway were then extracted and designated as Candidate Ferroptosis Gene Set 1 (cd_Ferr_geneset1), comprising a total of 41 genes (**Supplementary Table 2**).

### Subclusters of HCC cohort based on FRGs and PCA analysis

Molecular subtyping of patients with HCC was performed using FRGs-set1 through consensus clustering, utilizing the “ConsensusClusterPlus” R package. To ensure the robustness and stability of the identified subtypes, a consensus matrix was generated by repeating the clustering algorithm 100 times. For each iteration, a distance matrix was computed using 1 minus the absolute Pearson correlation coefficient (1-|Pearson|), and agglomerative hierarchical clustering (method = “hc”) with average linkage was applied. The optimal number of stable clusters (k) was determined by evaluating the consensus cumulative distribution function (CDF) and tracking the cluster consensus index across a predefined range of k values (31). Principal component analysis (PCA) was subsequently performed to assess classification performance using the “ggplot2” and “ggbiplot” package. At K = 2, TCGA HCC samples were stratified into two distinct groups (designated as ferr.clusterA and ferr.clusterB), which exhibited a statistically significant difference in overall survival (OS). Subsequently, DEGs between these two ferroptosis-related subtypes were identified using the “limma” package with thresholds of |FC| > 1.5 and an adjusted *p< 0.05*. This analysis yielded 4,033 DEGs, which were defined as Candidate Ferroptosis Gene Set 2 (cd_Ferr_geneset2, **Supplementary Table S3**).

### Weighted gene co-expression network analysis and identification of HCC hub genes

The gene sets FRGs-set1, cd_Ferr_geneset1, and cd_Ferr_geneset2 were merged and subjected to weighted gene co-expression network analysis (WGCNA). The resulting co-expression network exhibited scale-free topology, as indicated by a strong negative linear correlation between log(k) and log(P(k)) (correlation coefficient > 0.8). To ensure scale-free network properties, the optimal soft-thresholding power β was set to 8. The expression matrix was first transformed into an adjacency matrix and then into a topological overlap matrix (TOM). Based on the TOM, genes were clustered using average-linkage hierarchical clustering. Dynamic tree cutting was applied with a minimum module size of 30 genes. After initial module detection, module eigengenes were calculated and modules were further merged by clustering with a height cutoff of 0.25, yielding six consensus modules. Gene significance (GS) was then computed for each module, where a higher GS indicating stronger association with overall survival (OS). Additionally, Pearson correlation coefficients between module eigengenes and OS were calculated, where a stronger correlation reflected a more robust link to OS. Based on both GS and correlation analyses, the turquoise module was selected as the key module. Protein–protein interaction (PPI) network analysis of turquoise-module genes identified 205 core genes under the criterion of degree > 12. Meanwhile, 138 core genes were selected from the turquoise module using thresholds of module membership (MM) > 0.7 and GS > 0.15. The intersection of these two gene sets comprised 47 genes, which were defined as HCC hub genes (**Supplementary Table S4**).

### Construction of multi-factor regulatory networks

Transcriptional regulation data were retrieved from the TRRUST database (https://www.grnpedia.org/trrust/) to identify transcription factors (TFs) targeting the HCC-hub genes. Regarding miRNA-mRNA targeting relationships, the high-confidence interactions were extracted from TargetScan (http://www.targetscan.org/vert_72/), miRTarBase (https://mirtarbase.cuhk.edu.cn/), and miRDB (http://mirdb.org/). Only miRNAs predicted to target the HCC-hub genes by all three databases were retained as high-confidence regulatory miRNAs (filtered miRNA set). Long non-coding RNA (lncRNA)-miRNA interactions were subsequently obtained from Starbase (https://rnasysu.com/encori/). LncRNA interacting with the filtered miRNA set were incorporated to construct a multi-layer regulatory network. Finally, an integrated TF-mRNA-miRNA-lncRNA regulatory network was reconstructed and visualized using cytoscape (v3.9.1).

### Construction and validation of ferroptotic lenvatinib-related prognostic signature

To identify prognostic genes, univariate Cox regression analysis was performed on the HCC hub genes. Subsequently, key genes were selected via LASSO regression to construct a prognostic model (FLRPS). Patients were stratified into high-risk and low-risk groups based on the cohort-specific median risk score. The predictive performance of the model was evaluated using Kaplan-Meier analysis, and time-dependent ROC curves for 0.5-, 1-, and 3-year survival were generated using the R package “survivalROC”. The risk score for each patient was calculated as follows: Score = Σ(mRNA expression × corresponding regression coefficient).

### Copy number variation analysis

The CNV profiles across all samples were identified using the GISTIC 2.0 algorithm based on SNP6 CopyNumber segment data. The analysis was performed with the following parameters: a q-value threshold of< 0.05 was applied to define significant CNV, and a confidence level of 0.90 was used to define peak regions. These analyses were conducted via the GISTIC 2.0 module within the GenePattern platform (https://www.genepattern.org/) developed by the Broad Institute.

### Development of a nomogram

A prognostic nomogram integrating the FLRPS risk score with established clinicopathological variables was devised to provide individualized predictions of 1-, 3-, and 5-year overall survival. The discriminatory capacity of the nomogram was quantified using time-dependent ROC analysis, and its calibration was graphically evaluated with calibration curves. All statistical analyses were performed using the “rms” package in R.

### Immunohistochemistry

Formalin-fixed, paraffin-embedded tissue sections were deparaffinized in xylene and rehydrated through a graded ethanol series. Antigen retrieval was performed in citrate buffer (pH 6.0) using microwave heating. Endogenous peroxidase activity was quenched with 3 % H_2_O_2_ for 15 min at room temperature, and non-specific binding was blocked with 10 % normal goat serum for 30 min. Sections were incubated overnight at 4 °C with primary antibodies diluted in antibody diluent. After washing with phosphate-buffered saline (PBS), HRP-conjugated secondary antibodies were applied for 1 h at room temperature. Signals were developed with 3,3’-diaminobenzidine (DAB), followed by hematoxylin counterstaining and dehydration. Quantification was performed by three independent blinded evaluators using a validated 12-point scoring system that integrated the percentage of immunopositive cells and staining intensity. The proportion of positive cells was scored as 0 (0–5 %), 1 (6–25 %), 2 (26–50 %), 3 (51–75 %), or 4 (>75 %). Intensity was graded as 0 (absent), 1 (weak), 2 (moderate), or 3 (strong). A composite score (range 0–12) was calculated by multiplying the proportion and intensity scores. The antibodies, their catalog numbers, and working dilutions used in this study are summarized as follows: CENPA (Proteintech, Cat. No. 26754-1-AP, 1:200); RAN (Proteintech, Cat. No. 82978-1-RR, 1:300); CDCA8 (Proteintech, Cat. No. 12465-1-AP, 1:300); KIF2C (Proteintech, Cat. No. 28372-1-AP, 1:150); YWHAB (Proteintech, Cat. No. 10936-1-AP, 1:100); HDAC2 (Proteintech, Cat. No. 12922-3-AP, 1:2000); DYNC1H1 (Proteintech, Cat. No. 12345-1-AP, 1:100); NRAS (Proteintech, Cat. No. 10724-1-AP, 1:200); Phospho-PI3 Kinase p85(Tyr458)/p55(Tyr199) (Affinity Biosciences, Cat# AF3242, 1:50); PI3 Kinase p85 (Proteintech, Cat No. 60225-1-lg, 1:500); Phospho-AKT (Ser473) (Proteintech, Cat No. 66444-1-lg, 1:200); Akt (Proteintech, Cat. No. 60203-2-lg, 1:200). For paired pre-treatment and resistant tissue samples, statistical comparisons of IHC scores were performed using the paired t-test. For pathway.

### Cell Counting Kit-8 assay

Cell viability was evaluated utilizing the CCK-8 (Dojindo Laboratories, Kumamoto, Japan). Stable YWHAB-knockdown Hep1–6 cells and their corresponding control cells were pretreated with 740Y-P (HY-P0175, MedChemExpress, New Jersey, USA, 30μM) for 24h (32). Subsequently the cells were plated in 96-well plates at a density of 3000 cells per well and cultured under standard conditions. Following cell attachment, the cells were treated with lenvatinib at various concentrations (0, 5, 10, 25, and 50 μM) for 96 hours. Following treatment, CCK-8 reagent was added to each well at 10% of the total medium volume, and the plates were incubated at 37 °C for 2 hours. The absorbance was measured at 450 nm using a microplate reader. Viability was normalized to that of untreated control cells and expressed as relative cell viability. Each experiment was repeated in three independent replicates. Intergroup comparisons were conducted using the unpaired two-tailed Student’s t-test. Data are shown as mean ± SEM.

### Western blotting

Cellular proteins were extracted using RIPA lysis buffer supplemented with a protease inhibitor cocktail. Protein concentrations were determined using a BCA protein assay kit. Equal quantities of protein were denatured, separated by sodium dodecyl sulfate-polyacrylamide gel electrophoresis, and subsequently electrophoretically transferred to polyvinylidene fluoride (PVDF) membranes (Millipore, Catalog # ISEQ00010). The membranes were blocked with 5% non-fat milk prepared in Tris-buffered saline containing 0.1% Tween-20 (TBST) for 1 hour at room temperature to minimize non-specific binding. Subsequently, the membranes were incubated overnight at 4 °C with primary antibodies. After washing with TBST, the membranes were incubated for 1 hour at room temperature with horseradish peroxidase (HRP)-conjugated secondary antibodies. Protein bands were visualized using an enhanced chemiluminescence (ECL) substrate (Thermo Scientific, Pierce) and detected via a chemiluminescence imaging system (Tanon T4600). The antibodies, their catalog numbers, and working dilutions used in this study are summarized as follows: YWHAB (Cell Signaling Technology, Catalog # 9636; dilution 1:1000); Beta Actin (Proteintech, Cat No. 66009-1-Ig; dilution 1:10000).

### Single-cell RNA sequencing and spatial transcriptomics analysis

The GSE299340 dataset was preprocessed using the “Seurat” package. Low-quality cells were excluded based on thresholds (< 250 or > 7000 expressed genes; >15% mitochondrial unique molecular identifiers). Mitochondrial, ribosomal, and hemoglobin genes were removed. Batch effects were corrected with Harmony. The top 2,000 variable genes were identified using FindVariableFeatures (default parameters). Cell clusters were generated via the “FindClusters” and “FindNeighbors” functions, visualized by Uniform Manifold Approximation and Projection (UMAP) method, and manually annotated. The DEGs between high/low YWHAB groups were identified using Seurat’s FindMarkers with Wilcoxon test (p.adj < 0.05). The cell lineage trajectory was established using Monocle2. Cell-cell interaction analysis was performed with the “CellChat” R package, employing the default CellChatDB ligand-receptor database following standard protocol. Specific interaction patterns between tumor cells and various cell types were inferred by detecting marker gene expression in tumor cells. For spatial transcriptomic validation, HCC Stereo-seq v1.3 transcriptome data were obtained from the SPATCH database (sample ID 11; https://spatch.pku-genomics.org/). YWHAB, GPC3, and AFP expression were visualized on identical spatial coordinates.

### *In vivo* xenograft model

All animal experimental procedures were strictly performed in accordance with the National Institutes of Health Guide for the Care and Use of Laboratory Animals and were approved by the Institutional Animal Care and Use Committee (IACUC) of Peking University Third Hospital (Approval No: SA2022001). Six-to-eight-week-old male BALB/c nude mice (body weight range: 18–22 g) were obtained from Vital River Laboratories, Beijing, China. All mice were housed under standard SPF conditions with a 12/12 h light/dark cycle, controlled temperature (22 ± 2 °C), and humidity (50 ± 10%).

To investigate whether YWHAB modulates lenvatinib response through ferroptosis, shRNA-YWHAB (sh-YWHAB) or shRNA-YWHAB control (sh-YWHAB ctrl) Hepa1–6 cells in the exponential growth phase were harvested, washed, and resuspended in sterile PBS. Each mouse was inoculated subcutaneously on the right dorsal flank with 5 x 10^6^ Hepa1–6 cells (sh-YWHAB or sh-YWHAB ctrl) in a 100 μL suspension. Tumor growth was monitored by measuring the length (L) and width (W) with digital calipers. Tumor volume (TV) was calculated using the formula: TV = (L×W²)/2. When tumor volumes reached approximately 100–150 mm³, the mice were randomly assigned to six groups (n = 5 per group): sh-YWHAB ctrl + PBS, sh-YWHAB + PBS, sh-YWHAB ctrl + lenvatinib, sh-YWHAB + lenvatinib, sh-YWHAB + lenvatinib + DMSO, and sh-YWHAB + lenvatinib + liproxstatin-1. The control group received oral gavage of vehicle (PBS) following the same schedule. The lenvatinib-treated groups received oral gavage of lenvatinib (HY-10981, MedChemExpress) at a dose of 4 mg/kg. The treatment regimen consisted of daily administration for five consecutive days, followed by a two-day drug holiday (33). This cycle was repeated once (resulting in a second five-day treatment period), for a total of 10 doses. After the treatment period, a 7-day observation period was implemented prior to sacrifice. Liproxstatin-1 (10 mg/kg) or DMSO (1%) was administered intraperitoneally every other day beginning on the first day of lenvatinib treatment (34). All mice received deep anesthesia via sodium pentobarbital (50 mg/kg, i.p.) prior to euthanasia by cervical dislocation.

To investigate whether YWHAB modulates lenvatinib mediated ferroptosis via PI3K-AKT pathway, the mice loading tumors were randomly assigned to three groups (n = 5 per group): sh-YWHAB ctrl + lenvatinib + DMSO (1%), sh-YWHAB + lenvatinib + DMSO (1%), and sh-YWHAB + lenvatinib + 740Y-P. 740Y-P (100 mg/kg/day) was injected intraperitoneally three times a week beginning on the first day of lenvatinib treatment (35).

### Invasion assay

To assess cell invasion ability *in vitro*, the transwell chamber inserts (8 µm pore size, Corning) were used. 200 µL matrigel (Corning, BD Biocoat) was coated onto the upper compartment of insert for 4 h at 37 °C. Hepa1–6 cells expressing sh-YWHAB or sh-YWHAB ctrl were randomly assigned to three treatment groups: sh-YWHAB ctrl + lenvatinib + 1% DMSO, sh-YWHAB + lenvatinib + 1% DMSO, and sh-YWHAB + lenvatinib + 30 µM 740Y-P (pretreatment for 24 h). Cells (1.0 × 10^4^) were resuspended in 200 µL of medium containing 1% FBS and 30 µM lenvatinib, and then seeded into the upper chamber. Medium supplemented with 10% FBS was placed in the lower chamber as a chemoattractant. After 24 h of incubation, non-invading cells were removed from the upper surface of the inserts. Invading cells on the lower surface were fixed with 4% paraformaldehyde (10 min, room temperature), stained with 0.1% crystal violet (30 min), and washed thrice with PBS. Images of the stained cells were captured using a microscope (Nikon, Japan, L300N/300ND). Each experiment was repeated in three independent replicates. Intergroup comparisons were conducted using the unpaired two-tailed Student’s t-test. Data are shown as mean ± SEM.

### Wound healing assay

The effect of YWHAB knockdown on cell migration was evaluated using a wound healing assay. Sh-YWHAB or sh-YWHAB ctrl Hepa1–6 cells were seeded into 6-well plates at a density of 6×10^5^ cells per well. The cells were then divided into three experimental groups: a control group (sh-YWHAB ctrl + lenvatinib + 1% DMSO), a YWHAB knockdown group (sh-YWHAB + lenvatinib + 1% DMSO), and a group with additional PI3K activation (sh-YWHAB + lenvatinib + 30 µM 740Y-P, pretreated for 24 h). Upon reaching 95–100% confluence, a sterile 200-µL pipette tip was used to scratch the cell. Following three washes with PBS, the medium was switched to serum-free medium containing 30 µM lenvatinib. Images of the wound were captured at 0 and 48 h post-scratch using a microscope (Nikon, L300N/300ND, Japan). Each experiment was repeated in three independent replicates. Intergroup comparisons were conducted using the unpaired two-tailed Student’s t-test. Data are shown as mean ± SEM.

## Results

### The flow chart of the analysis process

**Figure 1** outlined the analytical workflow. FRGs from FerrDb and published literature were compiled as FRGs-set1 (**Supplementary Table S1**). Differentially expressed genes (DEGs) between HCC and normal tissues underwent KEGG analysis, and the genes of the ferroptosis pathway identified from this analysis formed cd_Ferr_geneset1 (**Supplementary Table S2**). Consensus clustering based on FRGs-set1 stratified HCC into two subtypes, and the corresponding DEGs (cd_Ferr_geneset2, **Supplementary Table S3**) were identified. The union of cd_Ferr_geneset1, FRGs-set1, and cd_Ferr_geneset2 constituted the ferroptosis_geneset which was subjected to WGCNA to identify hub genes in HCC (**Supplementary Table S4**) for the construction of a regulatory network. Survival-related genes were screened via univariate Cox and LASSO regression. LRAGS were identified from GSE273819, GSE186191, and GSE252752. Using the intersection of the survival-related genes selected by LASSO and the LRAGS, the FLRPS was constructed and validated. The patients were stratified into high-risk and low-risk groups based on the FLRPS score for the analysis of mutations, the immune microenvironment, immunotherapy response, immune checkpoint expression, drug resistance, and gene set enrichment analysis (GSEA). Among the candidate genes (CENPA, RAN, CDCA8, KIF2C, YWHAB, HDAC2, DYNC1H1, and NRAS), the expression of the YWHAB was significantly upregulated in lenvatinib-resistant HCC, implicating its role in drug resistance. The effects of YWHAB on the phenotypes of HCC and the response to lenvatinib were validated *in vitro* and *in vivo*, and YWHAB was further demonstrated to modulate the PI3K-AKT pathway.

**Figure 1:**
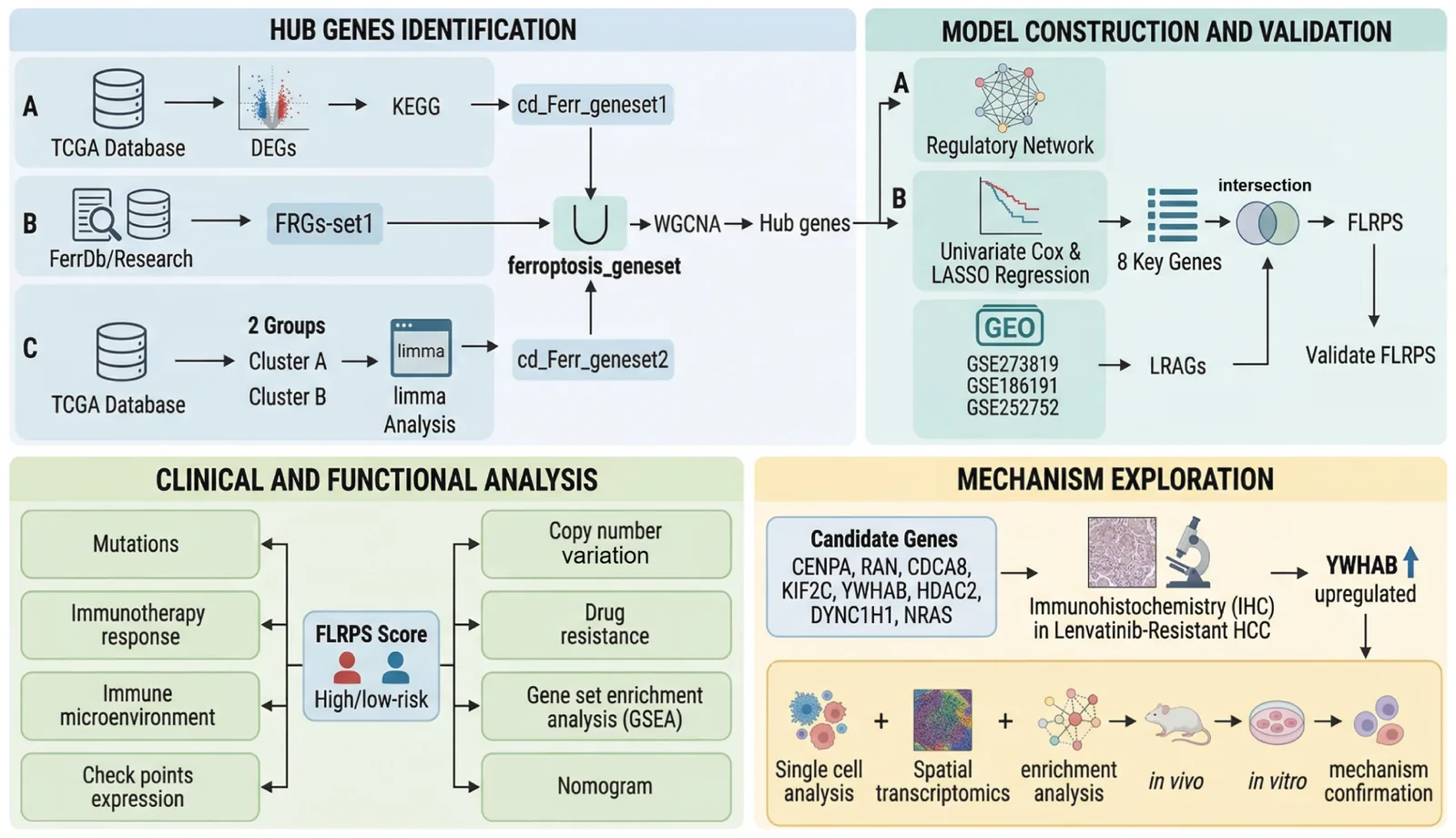
Flowchart of the study.

### Identification of the FRGs in HCC and weighted gene co-expression network analysis

In the comparison between HCC and normal tissues, 396 genes were downregulated and 998 were upregulated (**Supplementary Figure S1A**), and the top 20 most significantly altered genes are displayed in **Supplementary Figure S1B**. KEGG pathway enrichment analysis of these 1,394 DEGs revealed significant associations with multiple pathways, and the top 62 terms are visualized in **Figure 2A**. Among these, 41 ferroptosis-related genes were selected as candidate ferroptosis gene set 1 (cd_Ferr_geneset1). Consensus clustering based on FRGs-set1 stratified TCGA HCC samples into two stable subtypes (k=2), designated as Cluster A and Cluster B (**Supplementary Figures S1C, D**). The patients in Cluster A exhibited significantly better clinical outcomes than those in Cluster B (**Supplementary Figure S1E**). Between the two clusters, 4033 DEGs were identified (**Figure 2B**) and defined as candidate ferroptosis gene set 2 (cd_Ferr_geneset2). PCA further confirmed a clear separation between the two subgroups based on these DEGs (**Supplementary Figure S1F**).

**Figure 2:**
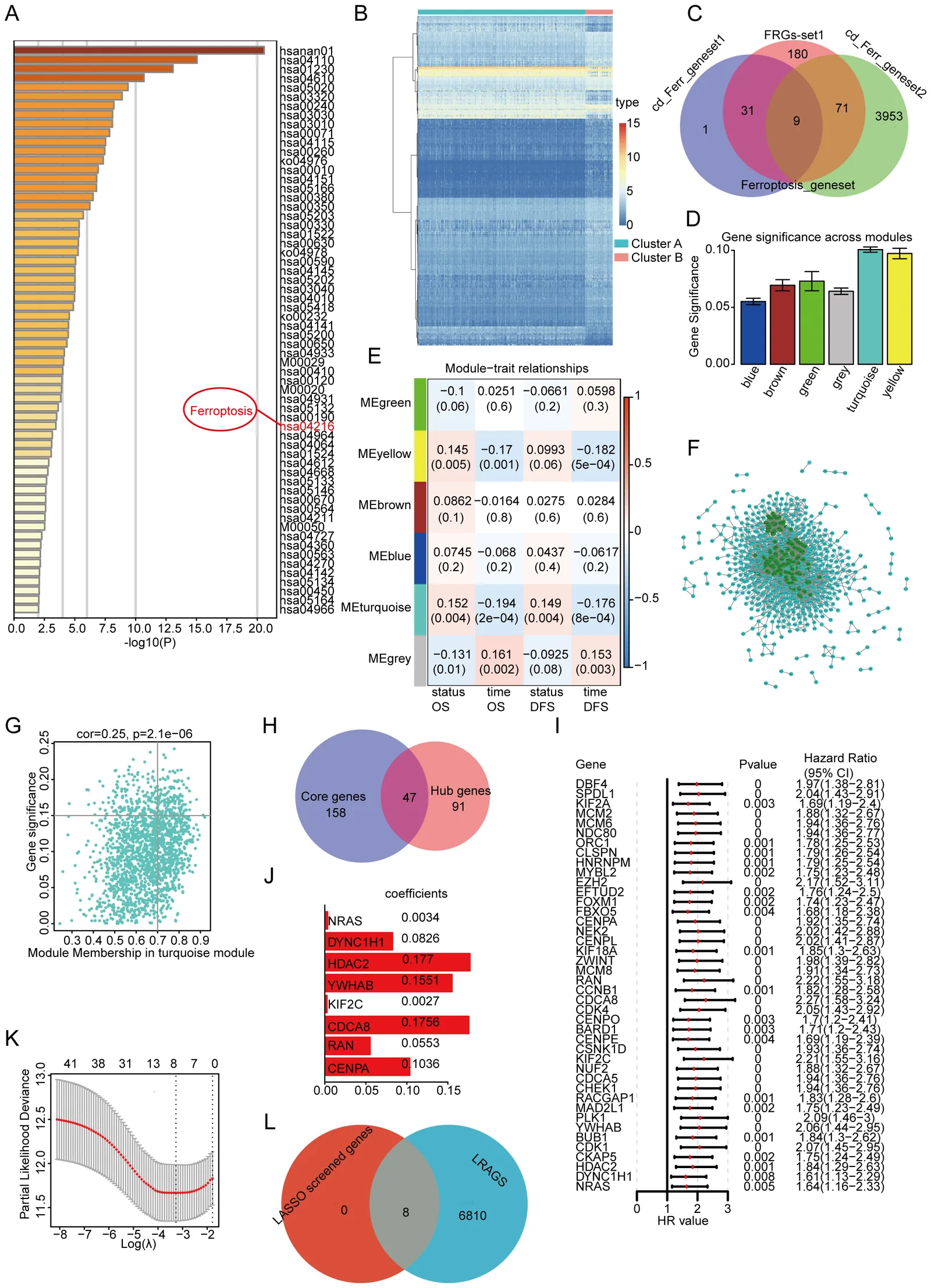
Construction of the FLRPS. **(A)** The top 62 KEGG terms. **(B)** The 4033 DEGs between the two clusters. **(C)** The combined gene set of FRGs-set1, cd_Ferr_geneset1, and cd_Ferr_geneset2. **(D)** Gene significance across modules. **(E)** The Pearson correlation coefficient between each module and the prognostic trait. **(F)** The protein-protein interaction network. **(G)** The module membership and gene significance in turquoise module. **(H)** The 47 HCC-hub genes. **(I)** Univariate Cox regression analysis of the 47 HCC-hub genes. **(J, K)** LASSO regression analysis. **(L)** The intersection of LASSO-selected genes and LRAGS. Abbreviations: DEGs, differential expression genes; WGCNA, weighted gene co-expression network analysis; LRAGS, lenvatinib resistance associated genes set; FLRPS, ferroptotic lenvatinib-related prognostic signature; GSEA, gene set enrichment analysis; ROC, receiver operating characteristic.

Subsequently, the union of cd_Ferr_geneset1, FRGs-set1, and cd_Ferr_geneset2 was generated and designated as ferroptosis_geneset (**Figure 2C**). Based on ferroptosis_geneset, WGCNA was performed using a soft-thresholding power (β) of 8, identifying six distinct co-expression modules (**Supplementary Figure S1G, H**). The gene significance (GS) for each module reflected its prognostic relevance to overall survival (OS), with a higher GS indicating stronger association (**Figure 2D**). The larger pearson correlation coefficient between each module and the prognostic trait further highlighted the greater clinical importance of the module (**Figure 2E**). Based on GS and correlation results, the turquoise module exhibited the strongest association with HCC. A protein-protein interaction network constructed from the genes of the turquoise module (**Figure 2F**) revealed 205 core genes (degree > 12). Additionally, 138 hub genes were selected from the turquoise module using criteria of module membership (MM) > 0.7 and GS > 0.15 (**Figure 2G**). The intersection of these core and hub genes yielded 47 HCC-hub genes (**Figure 2H**), which were utilized to construct a multifactorial regulatory network (**Supplementary Figure S1I**).

### Construction and validation of the FLRPS

Univariate Cox regression analysis of the 47 HCC hub genes identified 42 prognostic genes, including NRAS, CDCA8, RAN, and KIF2C, the higher expression of which correlated with poorer outcomes (**Figure 2I**, **Supplementary Figures S1J-M**). Subsequently, this set was further refined by LASSO regression to yield eight key prognosis-associated genes (RAN, CDCA8, KIF2C, NRAS, CENPA, YWHAB, HDAC2, and DYNC1H1, **Figures 2J, K**). DEGs from GSE186191, GSE273819, and GSE252752 were merged into LRAGS comprising 6818 genes (**Supplementary Figure S1N**). Notably, all eight genes selected via LASSO were present in the LRAGS (**Figure 2L**) and were utilized to construct the FLRPS (Risk Score=0.103626156*CENPA+0.055317101*RAN+0.175623707*CDCA8 + 0.002724613*KIF2C+0.155130535*YWHAB+0.176983779*HDAC2 + 0.082630248*DYNC1H1 + 0.003444740*NRAS). Using the median risk score (2.076101) as cutoff, patients were stratified into high-risk and low-risk groups, and analyses of risk score distribution and survival status confirmed higher scores and mortality in the high-risk group (**Figure 3A**). Expression heatmap revealed elevated levels of all 8 signature genes in high-risk patients (**Figure 3B**). Kaplan-Meier analysis demonstrated significantly worse survival for the high-risk patients (**Figure 3C**). Time-dependent ROC curves yielded area under the curve (AUC) values of 0.74 (0.5-year), 0.78 (1-year), and 0.70 (3-year) for the prediction of OS, demonstrating the robust predictive performance of the signature (**Figure 3D**). To further validate the prognostic accuracy of the FLRPS, risk scores were calculated using the same formula in two external cohorts. In the International Cancer Genome Consortium (ICGC) cohort (232 HCC patients), stratification by the median risk score (2.463143) again revealed upregulation of the eight signature genes (**Supplementary Figures S2A, B**) and significantly shorter OS (**Supplementary Figure S2C**) in the high-risk patients. Time-dependent ROC analysis yield AUCs of 0.73, 0.79, and 0.80 for 0.5-year, 1-year, and 3-year OS, respectively (**Supplementary Figure S2D**). Similarly, in the GSE14520 cohort (221 HCC patients), the high-risk patients (cutoff: 5.226997) exhibited significantly shorter OS (**Supplementary Figure S2E**), with corresponding AUCs of 0.66, 0.67, and 0.62 for 0.5-year, 1-year, and 3-year OS, respectively (**Supplementary Figure S2F**).

**Figure 3:**
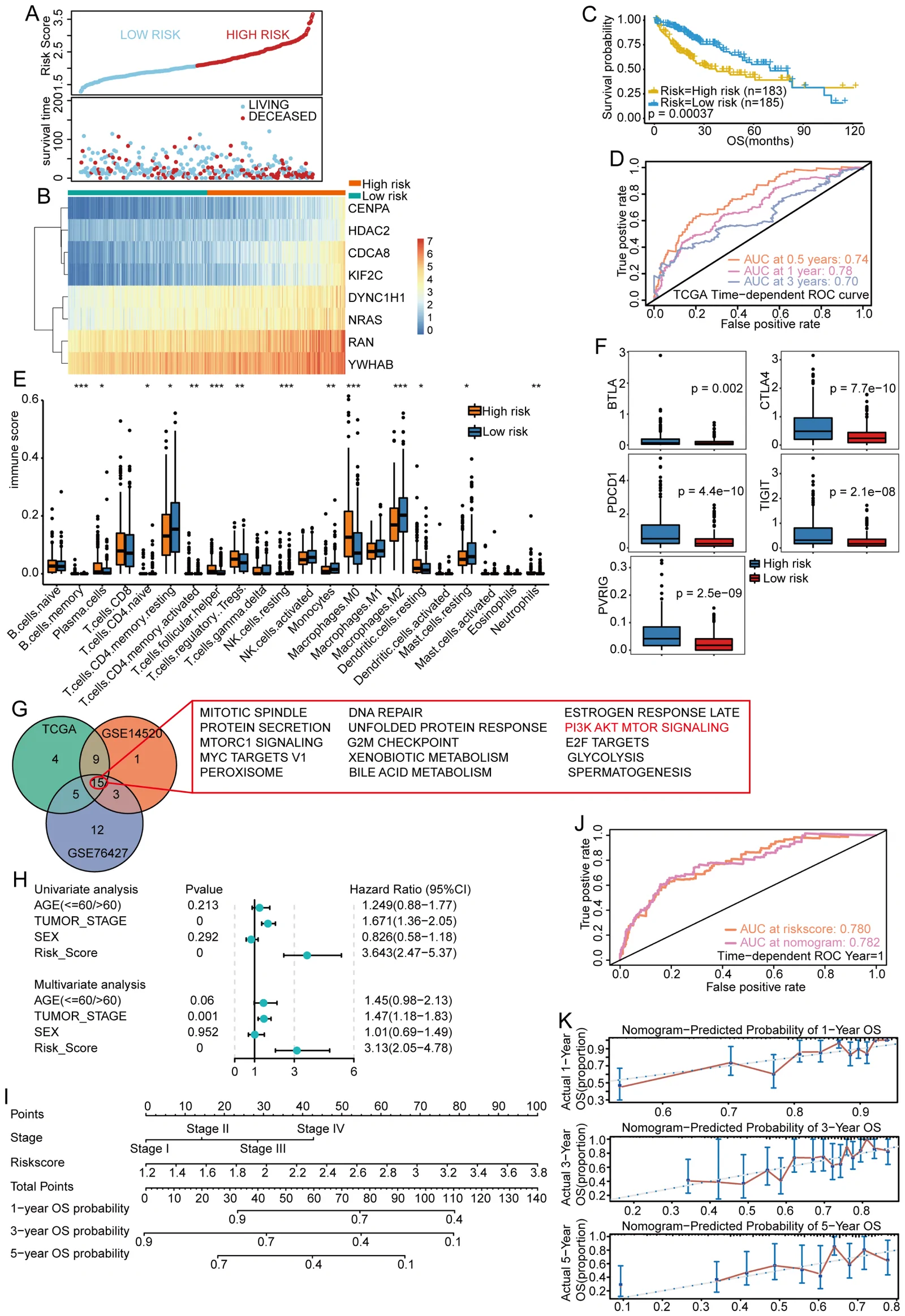
Validation of the FLRPS and construction of the nomogram. For patients with HCC from the TCGA cohort in the high-risk and low-risk groups, the following analyses were performed: **(A)** the risk score distribution and survival status; **(B)** the expression levels of RAN, CDCA8, KIF2C, NRAS, CENPA, YWHAB, HDAC2, and DYNC1H1; **(C)** Kaplan–Meier survival analysis; **(D)** and ROC analysis. **(E)** CIBERSORT analysis. **(F)** The expression of key immune checkpoint molecules between the risk groups in patients with HCC from the TCGA cohort. **(G)** Signaling pathways associated with the FLRPS. **(H)** Univariate and multivariate Cox regression analyses. **(I)** Construction of the nomogram integrating the FLRPS score and tumor stage. **(J)** ROC analysis of the nomogram. **(K)** Calibration curves for the nomogram. (*P< 0.05; **P< 0.01; ***P< 0.001). AUC, area under the curve; OS, overall survival.

Furthermore, the associations between the FLRPS and the clinicopathological features of the patients with HCC from the TCGA dataset were analyzed. The high-risk group exhibited significantly worse OS across most subgroups (**Supplementary Figure S2G**). Although the difference was not statistically significant (*P>0.05*) in Grade 3–4 or female patients, trends toward poorer survival were observed in high-risk individuals within these subgroups, which further validated the stability of the FLRPS. Consistently, higher risk scores were associated with advanced tumor grades and AJCC stages (**Supplementary Figures S2H, I**).

The predictive performance of the FLRPS was further evaluated in gastrointestinal cancers from the TCGA database, including esophageal, gastric, colon, and rectal adenocarcinomas, by calculating the risk scores from their expression profiles. The samples were divided into high-risk and low-risk groups based on the median FLRPS scores, and OS was compared between groups. The results indicated that FLRPS did not predict prognosis in these cancers (**Supplementary Figure S2J**), supporting the specificity of FLRPS for HCC.

Based on bulk RNA-seq data, the Single-cell and Spatially-resolved Cancer Resources (SCAR, http://scaratlas.com/SCAR_Survival.html) was leveraged to confirm the adverse prognostic impact of CENPA, RAN, KIF2C, HDAC2, DYNC1H1, and CDCA8, (**Supplementary Figure S3A**), thereby reinforcing the prognostic relevance of the eight FLRPS genes in HCC. Correspondingly, each of these eight genes exhibited higher expression in the HCC tissues from the TCGA dataset compared with the normal tissues (**Supplementary Figure S3B**).

### Immune infiltration, mutational landscape, and pathway enrichment in different FLRPS risk groups

To explore the association between the scores of the FLRPS and the tumor immune microenvironment, the immune subtypes of HCC were defined as C1 (wound healing), C2 (IFN-γdominant), C3 (inflammatory), and C4 (lymphocyte depleted). While the C3 subtype exhibited the best prognosis, C2 and C1 subtype demonstrated less favorable outcomes, and the C4 subtype presented the poorest prognosis (36). Correspondingly, the results of this study indicated that the lowest risk scores of the FLRPS were observed in the C3 subgroup, whereas higher risk scores were observed in the C1 and C2 subgroups, which is in accordance with the predictive capacity of the FLRPS (**Supplementary Figure S4A**).

To assess how the FLRPS regulates tumor immunogenicity, key determinants, including mutation load, neoantigen load, chromosomal instability, and the stemness index, were examined. FLRPS scores correlated positively with chromosomal instability (CNA fraction altered), and the mRNAsi-based stemness index (mRNAsi) (**Supplementary Figure S4B**). Tumors characterized by chromosomal instability are considered immunologically “cold,” which is marked by T-cell exhaustion and the infiltration of tumor-associated macrophages (TAMs). The high mRNAsi correlates with reduced immune and stromal components. Consequently, high-risk HCC patients may derive limited benefits from immunotherapy.

To examine associations between FLRPS scores and immune infiltration, CIBERSORT analysis was utilized to quantify 22 immune cell types across risk groups (**Figure 3E**). High-risk patients exhibited elevated levels of memory B cells, plasma cells, activated CD4^+^ memory T cells, follicular helper T cells, regulatory T cells, M0 macrophages, resting dendritic cells, and neutrophils. In contrast, the levels of naive CD4^+^ T cells, resting CD4^+^ memory T cells, resting NK cells, monocytes, M2 macrophages, and resting mast cells were significantly lower in this group. These findings suggest that FLRPS contributed to a remodeled immune landscape. Additionally, high-risk HCC patients exhibited elevated expression of key immune checkpoint molecules, including BTLA, CTLA4, PDCD1, TIGIT, and PVRIG, compared to low-risk patients (**Figure 3F**).

Subsequently, the differences in somatic mutations between the high-risk and low-risk groups were analyzed using the “maftools” R package. Waterfall plots displayed the top 14 most frequently mutated genes in the high-risk and low-risk groups in **Supplementary Figure S4C**. As demonstrated, the mutation genes and mutation frequencies differed between these two groups. The high-risk group exhibited a significantly higher mutation frequency for TP53 compared with the low-risk group (46% vs. 14%). Conversely, the mutation frequency of CTNNB1 was lower in the high-risk group than that in the low-risk group (20% vs. 35%). **Supplementary Figure S4D** displays the landscape of the regions of copy number variations (CNVs) associated with hepatocarcinogenesis across the groups. The high-risk group exhibited a significantly higher frequency of CNVs than the low-risk group (**Supplementary Figure S4E**).

To identify the signaling pathways linked to the FLRPS, GSEA was conducted using the DEGs from the risk-stratified groups. The TCGA and GSE14520 cohorts revealed 33 and 28 enriched HALLMARK pathways, respectively (**Supplementary Figures S5A, B**). The patients in the GSE76427-HCC cohort were also stratified based on the FLRPS, which confirmed that the high-risk patients experienced poorer clinical outcomes (**Supplementary Figure S5C**). DEGs from this cohort enriched 35 HALLMARK pathways (**Supplementary Figure S5D**). The intersection of the significant pathways from all three analyses yielded 15 common ones (e.g., PI3K-AKT-MTOR signaling), indicating the involvement of these pathways in the progression of HCC mediated by the FLRPS (**Figure 3G**).

### The correlation of the FLRPS and therapies

To investigate the correlation between immunotherapy outcomes and the scores of the FLRPS, the risk score for each sample was calculated using expression profile data from the IMvigor210CoreBiologies dataset (348 samples, including 298 samples with available data on immunotherapy outcomes). Subsequently, the surv_cutpoint function from the R package survminer was employed to determine the optimal cutoff value for classification. Based on the optimal cutoff threshold (cutoff = 3.054422), the 298 samples were divided into high-risk and low-risk groups. Notably, among patients with stable disease (SD) and progressive disease (PD), those classified as high-risk demonstrated poorer clinical outcomes (**Supplementary Figure S6A**).

To assess the predictive efficacy of the FLRPS for multi-target tyrosine kinase inhibitor (mTKI) therapy in HCC, the GSE109211 dataset was analyzed. A significant difference was observed between the high-risk and low-risk groups (Fisher’s exact test, *P=3.186e-06*), with a non-responder rate of 97% in the high-risk group compared to 41% in the low-risk group (**Supplementary Figure S6B**). The non-responders exhibited significantly higher FLRPS scores than the responders (**Supplementary Figure S6C**). ROC analysis further demonstrated high predictive reliability for the response to mTKI therapy, yielding an AUC of 0.930 (**Supplementary Figure S6D**).

Furthermore, the association between the FLRPS and the efficacy of chemotherapy in HCC was investigated. The low-risk group exhibited significantly higher IC_50_ values for doxorubicin and cisplatin (**Supplementary Figure S6E**).

These exploratory results suggest that the FLRPS may be associated with responses to immunotherapy, mTKI therapy, and chemotherapy. However, these analyses require further validation and are not definitive for treatment selection.

### Construction and analysis of the nomogram for HCC patients

Univariate and multivariate Cox regression analyses identified both the FLRPS score and tumor stage as independent prognostic factors for OS (**Figure 3H**). Subsequently, a nomogram integrating these two factors was constructed (**Figure 3I**). Compared with the FLRPS alone (AUC of 0.780), the nomogram demonstrated improved predictive accuracy, achieving an AUC of 0.782 for the prediction of 1-year OS (**Figure 3J**). Furthermore, calibration curves indicated strong agreement between the predicted and observed probabilities for 1-year, 3-year, and 5-year OS (**Figure 3K**).

### YWHAB promoted lenvatinib resistances in HCC

To further assess the influence of genes related to the FLRPS on lenvatinib resistance, IHC analysis was performed for eight proteins (RAN, CDCA8, KIF2C, NRAS, CENPA, YWHAB, HDAC2, and DYNC1H1) using tumor tissues from four patients with HCC, which were collected both before lenvatinib treatment and after the development of resistance. The results revealed a significant increase exclusively in the protein levels of YWHAB in the post-resistance samples compared with the pre-treatment samples, whereas no significant changes were observed for the other seven proteins (**Figures 4A, B**; **Supplementary Figures S7**, **S8A**).

**Figure 4:**
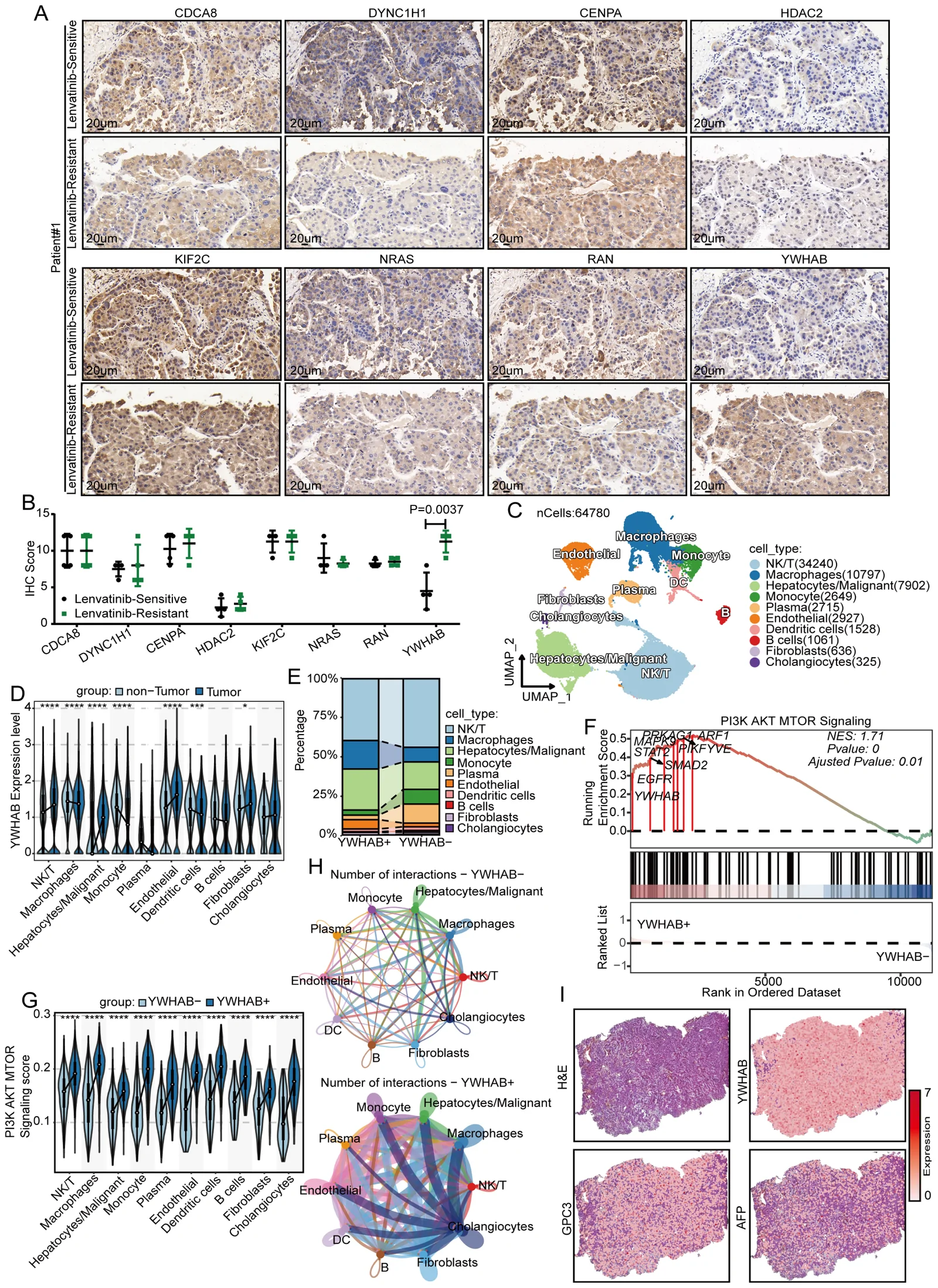
Single-cell transcriptomics and spatial transcriptomics of HCC. **(A, B)** IHC for CDCA8, DYNC1H1, CENPA, HDAC2, KIF2C, NRAS, RAN, and YWHAB in the tumor tissues of patients with HCC, with or without lenvatinib resistance. **(C)** The cell types of GSE299340. **(D)** Expression levels of YWHAB across different cell types. **(E)** Percentages of different cell types in YWHAB- and YWHAB+ groups. **(F)** Activation of the PI3K-AKT-mTOR pathway in the high YWHAB expression state. **(G)** The PI3K-AKT-MTOR signaling scores across different cell types. **(H)** Results of the CellChat analysis. **(I)** Spatial plots demonstrating prediction scores of YWHAB in the tumor region (corroborated by H&E staining, and GPC3 and AFP expression) within the SPATCH dataset. (*P< 0.05; ***P< 0.001; ****P< 0.0001). For the paired IHC analyses, comparisons were performed using the paired t-test. Data are shown as mean ± SEM and error bars represent SEM. H&E, hematoxylin-eosin staining; B, B cells; DC, Dendritic cells; NES, Normalized Enrichment Score; SEM, Standard Error of the Mean.

Additionally, single-cell transcriptomics identified 10 cell clusters (**Figure 4C**). YWHAB was highly expressed in HCC cells (**Figure 4D**). Compared to the YWHAB-low expression (YWHAB-) group, the YWHAB-high expression (YWHAB+) group exhibited a higher proportion of HCC cells and endothelial cells, a condition that contributes to the formation of an immunosuppressive microenvironment (**Figure 4E**). GSEA of DEGs between YWHAB+ and YWHAB- HCC cells revealed significant activation of the PI3K-AKT-MTOR pathway in the YWHAB-high state (**Figures 4F, G**). Results from CellChat demonstrated that interactions among immune cells and HCC cells were markedly enhanced and more frequent under YWHAB-high conditions (**Figure 4H**). As depicted, YWHAB was prominently localized within the core region of the tumor (identified by GPC3 and AFP, **Figure 4I**), a finding that was further corroborated by the SPATCH dataset.The role of YWHAB in the response to lenvatinib was further validated using a xenograft model. Hepa1–6 cells with YWHAB knockdown (shRNA-YWHAB, sh-YWHAB, **Figure 5A**) and their negative control cells (shRNA-YWHAB ctrl, sh-YWHAB ctrl) were injected subcutaneously into the flanks of mice. Downregulation of YWHAB enhanced lenvatinib-induced suppression of proliferation of Hepa1–6 cells *in vivo*, which was reversed by the ferroptosis inhibitor (Liproxstatin-1, **Figures 5B–D**), suggesting that YWHAB modulates lenvatinib resistance through ferroptosis. To investigate the mechanism by which sh-YWHAB regulated lenvatinib-induced ferroptosis, the DEGs (top 50 shown in **Supplementary Figure S8B**) between the sh-YWHAB and sh-YWHAB ctrl Hepa1–6 cells were analyzed (**Figure 5E**). KEGG pathway analysis revealed that these DEGs were significantly enriched in the PI3K-AKT signaling pathway (**Figure 5F**). These results indicate that YWHAB may modulate lenvatinib-related-ferroptosis through the PI3K-AKT pathway.

**Figure 5:**
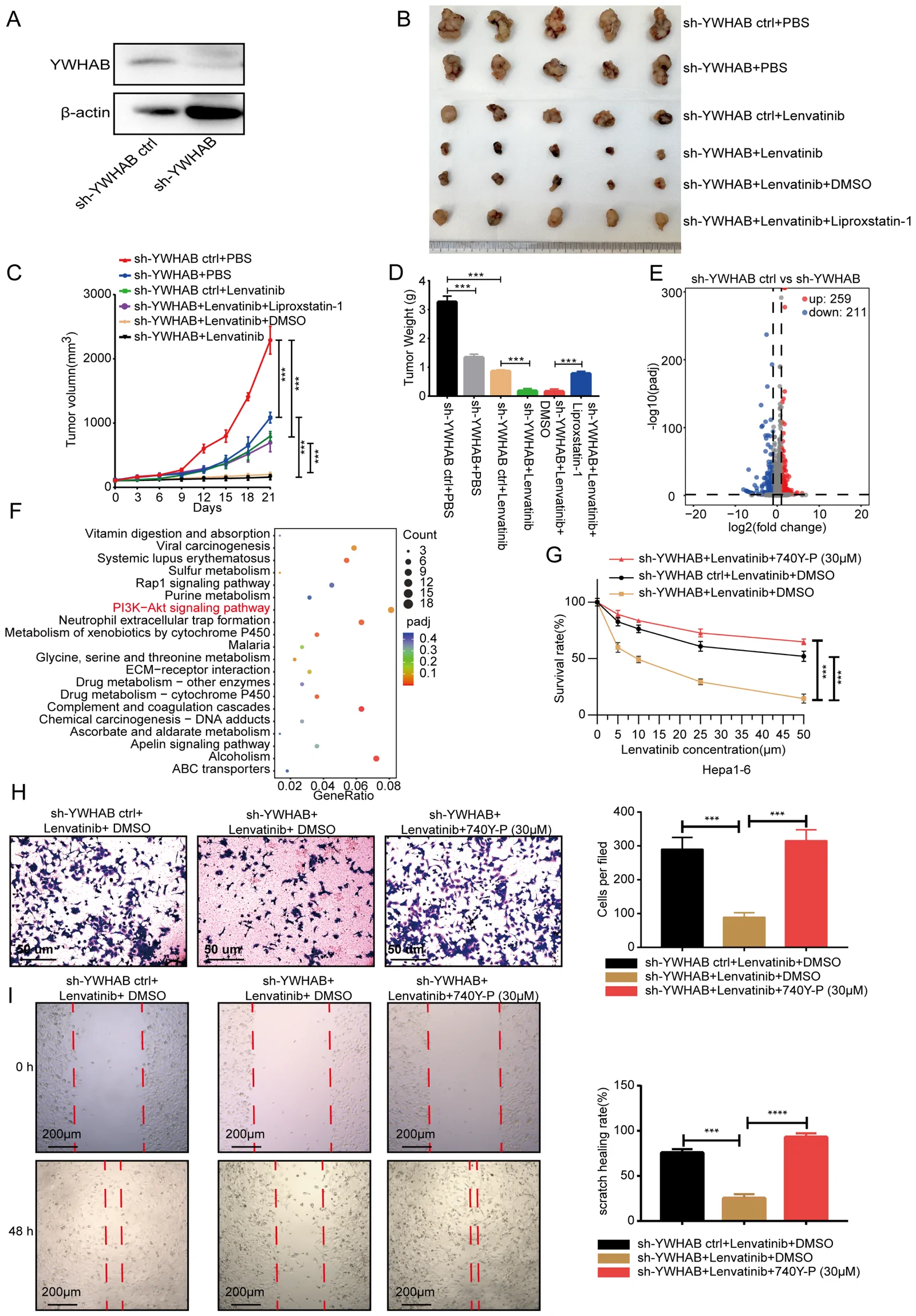
Sh-YWHAB enhances lenvatinib sensitivity in HCC through ferroptosis-related mechanisms via PI3K-AKT pathway inhibition. **(A)** Protein levels of YWHAB in the sh-YWHAB control and sh-YWHAB groups. **(B–D)** The volume and weight of HCC tumors with different levels of YWHAB with or without lenvatinib/liproxstatin-1 treatment. Lenvatinib was administered by oral gavage at 4 mg/kg for 5 days on/2 days off over two cycles; liproxstatin-1 was administered intraperitoneally at 10 mg/kg every other day. **(E)** The DEGs between sh-YWHAB and sh-YWHAB ctrl Hepa1–6 cells. **(F)** KEGG pathway analysis. **(G)** The effect of YWHAB on the growth of HCC cells following treatment with lenvatinib or 740Y-P, as evaluated by a CCK8 assay; **(H)** The effect of YWHAB on the invasive capacity of HCC cells following treatment with lenvatinib or 740Y-P, as evaluated by a Transwell assay. **(I)** The effect of YWHAB on the migratory ability of HCC cells following treatment with lenvatinib or 740Y-P, as evaluated by a wound-healing assay. (***P< 0.001; ****P< 0.0001). Intergroup comparisons for *in vitro* assays (n = 3 independent experiments) and tumor volume/weight (n = 5 mice per group) were conducted using the unpaired two-tailed Student’s t-test. Data are shown as mean ± SEM, and error bars represent SEM. sh-YWHAB, shRNA-YWHAB; sh-YWHAB ctrl, shRNA-YWHAB control; DMSO, dimethyl sulfoxide; SEM, Standard Error of the Mean.

In support of this hypothesis, *in vitro* experiments demonstrated that sh-YWHAB reduced the viability, invasive capacity, and migratory ability (measured by scratch healing rate) of Hepa1–6 cells upon lenvatinib treatment. Notably, these effects were reversed by 740 Y-P, an activator of the PI3K-AKT pathway (**Figures 5G–I**). *In vivo*, 740 Y-P was further shown to reverse the sensitization of Hepa1–6 cells to lenvatinib induced by the knockdown of YWHAB (**Figures 6A–C**). IHC analysis also confirmed that sh-YWHAB downregulated the protein levels of p-PI3K, p-AKT, GPX4, and Ki-67, whereas 740 Y-P counteracted these effects (**Figure 6D**). These findings suggest that the knockdown of YWHAB promotes lenvatinib-induced ferroptosis via the inhibition of the PI3K-AKT pathway.

**Figure 6:**
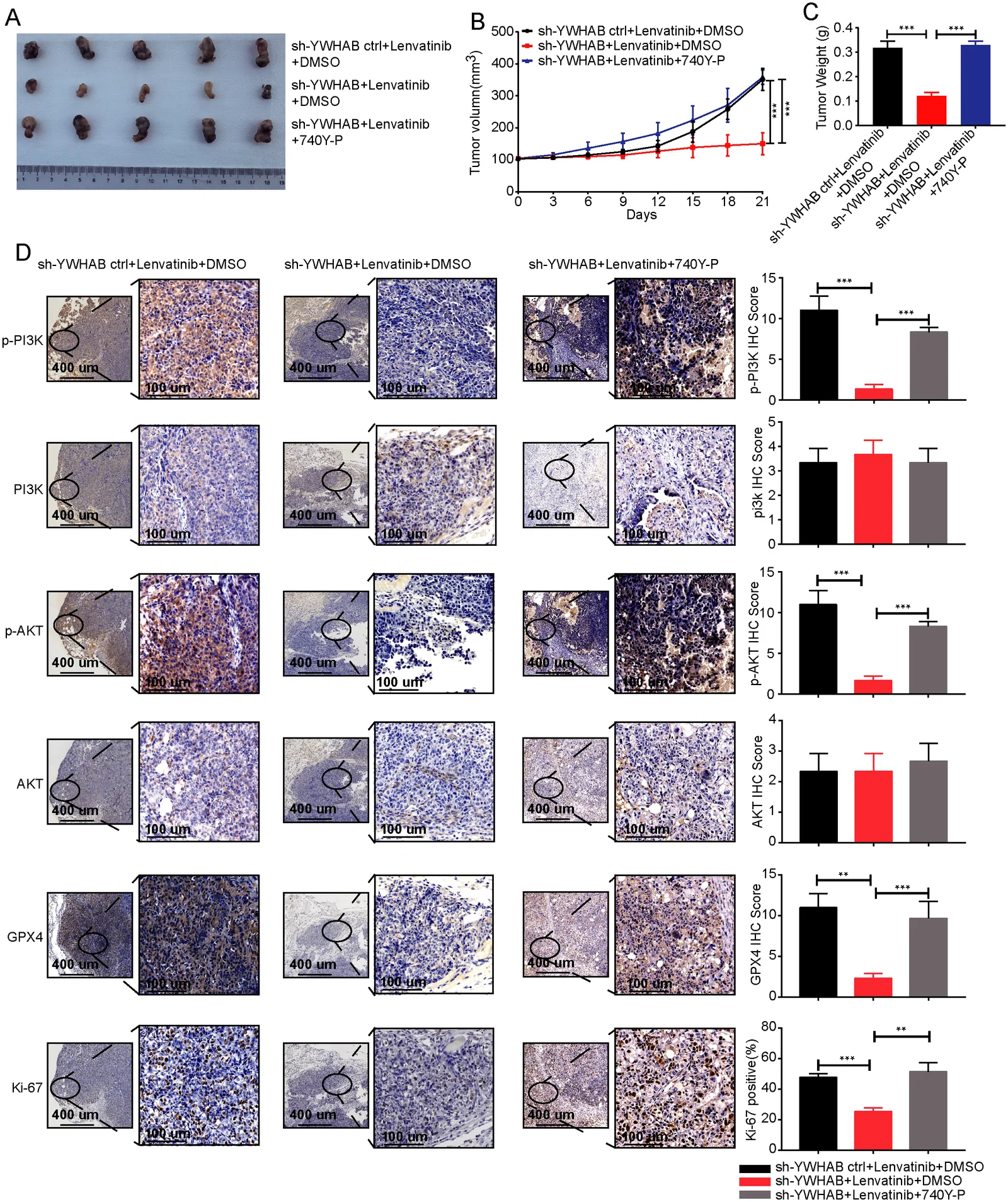
Sh-YWHAB enhances lenvatinib sensitivity in HCC *in vivo* through ferroptosis-related mechanisms via PI3K-AKT pathway inhibition. **(A–C)** The volume and weight of HCC tumors with different levels of YWHAB following treatment with Lenvatinib/740Y-P. **(D)** The effect of YWHAB on the protein levels of p-PI3K, PI3K, p-AKT, AKT, GPX4, and Ki-67 in HCC tumors following treatment with lenvatinib/740Y-P. Lenvatinib was administered by oral gavage at 4 mg/kg for 5 days on/2 days off over two cycles; 740Y-P was administered intraperitoneally at 100 mg/kg three times per week. (**P< 0.01; ***P< 0.001). Tumor volume, tumor weight, and IHC quantifications were analyzed by unpaired two-tailed Student’s t-test (n = 5 mice per group). Data are shown as mean ± SEM, and error bars represent SEM. sh-YWHAB, shRNA-YWHAB; sh-YWHAB ctrl, shRNA-YWHAB control; DMSO, dimethyl sulfoxide; IHC, immunohistochemistry; SEM, Standard Error of the Mean.

## Discussion

Resistance to lenvatinib represents a particularly pressing dilemma in the treatment of advanced HCC. Once progression occurs during lenvatinib-based first-line therapy, subsequent second-line options yield limited efficacy (37). Several studies investigated the mechanisms underlying lenvatinib resistance. Regulated by stress-activated STK24, the MASTL/YBX1/PAK4 axis induced lenvatinib resistance in HCC (38). Similarly, the p-MYH9/USP22/HIF-1α axis promoted lenvatinib resistance in HCC (44) and cancer-associated fibroblast-derived phosphoprotein 1 exerted a comparable effect (39). Furthermore, inhibition of EGFR reversed acquired lenvatinib resistance mediated by the STAT3–ABCB1 pathway in HCC (40), while NEK7-triggered EGFR phosphorylation at serine 1070 drove acquired lenvatinib resistance (41). Despite extensive research into these mechanisms, clinically validated ferroptosis-related predictive models based on lenvatinib resistance genes remained scarce. To address this gap, the present study constructed the FLRPS and explored the mechanism through which it drove lenvatinib resistance.

Several ferroptosis-related mechanisms have also been implicated in the response to lenvatinib in HCC. Lenvatinib triggers ferroptosis by upregulating LACTB (11), promoting HSD17B4-dependent peroxisomal β-oxidation (12), suppressing NRF2 activation and nuclear translocation (13), modulating Keap1 phosphorylation and the Keap1-NRF2 interaction (14), increasing HMOX1 and decreasing GPX4 (15), inhibiting the PPARγ-GPX4 axis (16), upregulating ALOX15 (17), and silencing FGFR4 to suppress system ${\bar{X}}_{c}$ (xCT) (18), whereas EZH2 suppresses ferroptosis via ACSL1 (19) and FSP1 promotes resistance by reducing intracellular and lipid ROS, Fe²^+^, and total iron (20). These observations support the central role of ferroptosis in the response to lenvatinib in HCC.In this study, eight genes comprising the FLRPS stratified HCC patients into high-risk and low-risk groups, demonstrating accuracy, reliability, and specificity as a predictive biomarker. Additionally, a nomogram model was constructed based on FLRPS and tumor stage, which exhibited favorable predictive performance. Recent research has established multiple ferroptosis-related prognostic models for HCC; specifically, ten recently published models based on ferroptosis-related genes demonstrated excellent predictive performance upon validation (**Supplementary Table S5**) (42–50). However, these signatures share common limitations. Primarily, genes associated with first-line therapeutic agents for HCC, including mTKIs, were not included in these signatures, leading to restricted clinical relevance. Furthermore, the absence of cytological and biological validation precluded the elucidation of the underlying molecular mechanisms. Comparing to existing predictive models, the FLRPS exhibited favorable predictive performance. Moreover, the FLRPS is the first ferroptotic signature developed based on lenvatinib resistance-associated genes. It primarily serves as a prognostic model for patients with HCC, demonstrating robust discrimination and prognostic performance. Exploratory analyses further suggested potential associations between the FLRPS and responses to mTKIs, immunotherapy, and chemotherapy. However, these treatment-response analyses are hypothesis-generating and require validation in independent, disease-appropriate cohorts. Thus, the FLRPS provides a clinically useful prognostic tool, whereas its utility for guiding therapeutic decision-making remains to be established in prospective studies.

The FLRPS consisted of eight genes—CENPA, RAN, CDCA8, KIF2C, YWHAB, HDAC2, DYNC1H1, and NRAS—all of which were upregulated in the high-risk group. CENPA is localized to centromeres and is essential for centromere integrity and function during mitosis (51). In HCC, CENPA has been reported to inhibit ferroptosis by promoting STMN1 transcription, thereby supporting tumor growth (52); this anti-ferroptotic direction is consistent with its association with the high-risk group. RAN participates in the NRF1/RAN/DLD protein complex that regulates CLTRN, and CLTRN enhances ferroptosis and radiosensitivity in HCC (53); however, whether RAN itself promotes or suppresses ferroptosis remains to be directly established. CDCA8 is a core component of the chromosomal passenger complex and is upregulated in multiple cancers (54), but a direct relationship between CDCA8 and ferroptosis has not been reported. KIF2C is a microtubule-depolymerizing kinesin that is overexpressed in multiple malignancies (63); recent evidence indicates that KIF2C suppresses ferroptosis, as KIF2C-deficient cancer cells upregulate ferroptosis genes and show increased erastin-induced ferroptosis (55), consistent with a ferroptosis-resistant direction in the high-risk group. HDAC2 belongs to the histone deacetylase family. In HCC, HDAC2 inhibition promotes sorafenib-induced ferroptosis, and the CTBP1/HDAC1/HDAC2 complex suppresses MAT1A to reduce ferroptosis (56, 57); a dual PI3K/HDAC inhibitor, BEBT-908, also induces immunogenic ferroptosis (58). DYNC1H1 mediates dynein transport along microtubules; its mRNA stability and overexpression have been associated with tumor progression in multiple cancers (59, 60), but a direct link between DYNC1H1 and ferroptosis has not been reported. NRAS encodes a RAS GTPase, and oncogenic RAS confers sensitivity to ferroptosis-inducing compounds (RAS-selective lethality) (61, 62); however, direct evidence linking NRAS upregulation to ferroptosis in HCC is limited. YWHAB encodes 14-3–3 family proteins, including 14-3-3β, which promote HCC cell migration and are associated with poor prognosis (63, 64). Our data indicate that YWHAB knockdown promotes lenvatinib-induced ferroptosis by modulating the PI3K-AKT pathway; however, this YWHAB-ferroptosis link has not been independently reported and should be regarded as an exploratory finding of the present study.

The present study revealed that YWHAB expression was elevated in HCC tissues and further upregulated upon acquisition of lenvatinib resistance. Knockdown of YWHAB significantly enhanced the therapeutic efficacy of lenvatinib in Hepa1–6 cells, as validated both *in vitro* and *in vivo*. This effect was accompanied by GPX4 downregulation and reversed by liproxstatin-1, consistent with a ferroptosis-related mechanism. Transcriptomic analysis indicated that YWHAB was significantly enriched in the PI3K-AKT signaling pathway. Notably, YWHAB knockdown reduced p-PI3K, p-AKT, Ki-67, and GPX4 levels, while suppressed cellular proliferation, invasion, and migration; these effects were reversed by the PI3K-AKT agonist 740Y-P. These findings expand the regulatory network of ferroptosis in lenvatinib resistance and identify YWHAB as a key mediator linking ferroptosis to drug resistance through modulation of the PI3K-AKT pathway. These results are consistent with previous reports of YWHAB dysregulation in various malignancies (65, 66) and addresses a critical gap in understanding its functional role in HCC. Consistent with our data, lenvatinib has been shown to inhibit the cystine/glutamate antiporter (xCT) and GPX4, thereby triggering lipid peroxidation and ferroptosis in HCC cells (18). More recent studies further support a GPX4-centered mechanism of lenvatinib sensitivity: the PPP1R15B/ATF4/GPX4 axis sustains lenvatinib-tolerant persister cells, and its inhibition re-sensitizes HCC cells to lenvatinib through ferroptosis (67), whereas AKR1B10 suppresses ferroptosis via the NQO1/GPX4 axis to promote acquired lenvatinib resistance (68).

Recently, several studies have reported the interaction between lenvatinib, ferroptosis and the TIME, which influences the therapeutic efficacy of HCC. Specifically, targeting SAMD1 sensitizes HCC to anti-PD-1/lenvatinib combination therapy through increased ferroptosis and enhanced immune activation (69). In the present study, the high-risk group exhibited significantly increased proportions of plasma cells, regulatory T cells, M0 macrophages, and neutrophils, alongside markedly decreased naive CD4+ T cells, resting memory CD4+ T cells, and resting mast cells. Existing evidence has linked high levels of plasma cells, Tregs, M0 macrophages, and tumor-associated neutrophils to poor OS (70–72). Conversely, low level of naive CD4+ T cells, resting memory CD4+ T cells, and resting mast cells are associated with a poor prognosis (73–75). Consistent with previous research, these findings suggest that the interaction between lenvatinib and ferroptosis may remodel the TIME.

The present study has several limitations. First, the bioinformatics analyses were mainly based on public databases, which may introduce inherent biases. Second, the IHC validation was conducted using only four pairs of tissue samples from HCC patients, which could result in potential bias. Third, ferroptosis was inferred from GPX4 downregulation and liproxstatin-1 rescue rather than direct measurements of lipid ROS, Fe²^+^, GSH/MDA, or transmission electron microscopy. Fourth, YWHAB functional validation was performed only in the mouse HCC cell line Hepa1-6, and the key findings have not been confirmed in a human HCC cell line. Fifth, the treatment-response and immunotherapy-related analyses were exploratory.

## Conclusions

In conclusion, this study established the FLRPS as an effective predictor of prognosis in HCC patients. Furthermore, our findings indicate that the knockdown of YWHAB activates ferroptosis by inhibiting the PI3K-AKT pathway in Hepa1–6 model, thereby enhancing the sensitivity of HCC to lenvatinib. These findings provide a novel decision-making tool for prognostic evaluation in HCC, while identifying a potential therapeutic target for reversing lenvatinib resistance.

## Data Availability

Publicly available datasets were analyzed in this study. These data can be found here: TCGA-LIHC (https://xenabrowser.net/datapages/); ICGC LIRI-JP (https://dcc.icgc.org/projects/LIRI-JP/); GEO (accession numbers GSE273819, GSE186191, GSE252752, GSE14520, GSE76427, GSE109211, and GSE299340 for scRNA-seq); IMvigor210 (http://research-pub.gene.com/IMvigor210CoreBiologies); FerrDb (http://www.zhounan.org/ferrdb/); and SPATCH (https://spatch.pku-genomics.org/, sample ID 11). All other data supporting the conclusions of this article are included within the article and its **Supplementary Material**.
