# Supplementary material for: Integrating ferroptosis and the tumor-immune gene mutations interface: a novel lenvatinib resistance prognostic signature with YWHAB-related immunotherapeutic correlates in hepatocellular carcinoma
Source: Front Immunol. 2026 Oct 29;17:1913042. doi: 10.3389/fimmu.2026.1913042 (PMC13755186; doi:10.3389/fimmu.2026.1913042)
Supplement: Supplementary file 2 [file Table1.docx]

**Supplementary Tables**

**Supplementary Table 1 ferroptosis-related genes set1**

| RPL8 | IREB2 | ATP5MC3 | CS | EMC2 | ACSF2 | NOX1 | CYBB | NOX3 | NOX4 | NOX5 | DUOX1 |
| --- | --- | --- | --- | --- | --- | --- | --- | --- | --- | --- | --- |
| DUOX2 | G6PD | PGD | VDAC2 | PIK3CA | FLT3 | SCP2 | TP53 | ACSL4 | LPCAT3 | NRAS | KRAS |
| HRAS | TF | TFRC | TFR2 | SLC38A1 | SLC1A5 | GLS2 | GOT1 | CARS1 | ALOX5 | KEAP1 | HMOX1 |
| ATG5 | ATG7 | NCOA4 | ALOX12 | ALOX12B | ALOX15 | ALOX15B | ALOXE3 | PHKG2 | ACO1 | G6PDX | ULK1 |
| ATG3 | ATG4D | BECN1 | MAP1LC3A | GABARAPL2 | GABARAPL1 | ATG16L1 | WIPI1 | WIPI2 | SNX4 | ATG13 | ULK2 |
| SAT1 | EGFR | MAPK3 | MAPK1 | BID | ZEB1 | DPP4 | CDKN2A | PEBP1 | SOCS1 | CDO1 | MYB |
| MAPK8 | MAPK9 | CHAC1 | MAPK14 | LINC00472 | PRKAA2 | PRKAA1 | ELAVL1 | BAP1 | ABCC1 | MIR6852 | ACVR1B |
| TGFBR1 | EPAS1 | HILPDA | HIF1A | IFNG | ANO6 | LPIN1 | HMGB1 | TNFAIP3 | TLR4 | ATF3 | ATM |
| YY1AP1 | EGLN2 | MIOX | TAZ | MTDH | IDH1 | SIRT1 | FBXW7 | PANX1 | DNAJB6 | BACH1 | LONP1 |
| PTGS2 | DUSP1 | NOS2 | NCF2 | MT3 | UBC | ALB | TXNRD1 | SRXN1 | GPX2 | BNIP3 | OXSR1 |
| SELENOS | ANGPTL7 | SLC7A11 | DDIT4 | LOC284561 | ASNS | TSC22D3 | DDIT3 | JDP2 | SESN2 | SLC1A4 | PCK2 |
| TXNIP | VLDLR | GPT2 | PSAT1 | LURAP1L | SLC7A5 | HERPUD1 | XBP1 | SLC3A2 | CBS | ATF4 | ZNF419 |
| KLHL24 | TRIB3 | ZFP69B | ATP6V1G2 | VEGFA | GDF15 | TUBE1 | ARRDC3 | CEBPG | SNORA16A | RGS4 | BLOC1S5-TXNDC5 |
| LOC390705 | EIF2S1 | KIM-1 | IL6 | CXCL2 | RELA | HSD17B11 | AGPAT3 | SETD1B | FTL | MAFG | IL33 |
| FTH1 | SLC40A1 | GPX4 | HAMP | HSPB1 | NFE2L2 | STEAP3 | DRD5 | DRD4 | MAP3K5 | SLC2A1 | SLC2A3 |
| SLC2A6 | SLC2A8 | SLC2A12 | GLUT13 | SLC2A14 | EIF2AK4 | TFAP2C | SP1 | HBA1 | NNMT | PLIN4 | HIC1 |
| STMN1 | RRM2 | CAPG | HNF4A | NGB | YWHAE | GABPB1 | AURKA | MIR4715 | RIPK1 | PRDX1 | MIR30B |
| AKR1C1 | AKR1C2 | AKR1C3 | RB1 | HSF1 | GCLC | SQSTM1 | NQO1 | MUC1 | MT1G | CISD1 | FANCD2 |
| FTMT | HSPA5 | HELLS | SCD | FADS2 | SRC | STAT3 | PML | MTOR | NFS1 | TP63 | CDKN1A |
| MIR137 | ENPP2 | FH | CISD2 | MIR9-1 | MIR9-2 | MIR9-3 | ISCU | ACSL3 | OTUB1 | CD44 | LINC00336 |
| BRD4 | PRDX6 | MIR17 | NF2 | ARNTL | JUN | CA9 | TMBIM4 | PLIN2 | MIR212 | Fer1HCH | AIFM2 |
| LAMP2 | ZFP36 | PROM2 | CHMP5 | CHMP6 | CAV1 | GCH1 | ACACA | ACSL1 | ACSL5 | ACSL6 | ATP5G3 |
| CFTR | CP | CRYAB | EGLN1 | FDFT1 | G3BP1 | GCLM | GSS | HMGCR | HSBP1 | ITGA6 | LOX |
| MAP1LC3B | MAP1LC3C | MDM2 | MIF | MYC | PCBP1 | PCBP2 | PRNP | SAT2 | SLC11A2 | SLC39A14 | SLC39A8 |
| SQLE | VDAC3 | CARS |  |  |  |  |  |  |  |  |  |

**Supplementary Table 2 Candidate Ferroptosis Gene Set 1 (cd_Ferr_geneset1)**

| HMOX1 | ATG5 | MAP1LC3C | CP | SLC39A8 | TF | ACSL3 | ACSL6 | GSS | SLC3A2 | FTMT |
| --- | --- | --- | --- | --- | --- | --- | --- | --- | --- | --- |
| VDAC2 | ATG7 | MAP1LC3B | PCBP1 | SLC39A14 | TFRC | TP53 | ACSL4 | GPX4 | SLC7A11 |  |
| VDAC3 | NCOA4 | MAP1LC3A | FTH1 | PCBP2 | STEAP3 | SAT2 | ACSL1 | ALOX15 | GCLC |  |
| CYBB | PRNP | MAP1LC3B2 | FTL | SLC40A1 | SLC11A2 | SAT1 | ACSL5 | LPCAT3 | GCLM |  |

**Supplementary Table3 Candidate Ferroptosis Gene Set 2 (cd_Ferr_geneset2)**

| NTS | ZDHHC23 | PSME3 | B3GNT7 | RAB3IL1 | TMX3 | PDCL3 | MAP4 | SCMH1 | STK39 | PPP1R18 | LRRC16A |
| --- | --- | --- | --- | --- | --- | --- | --- | --- | --- | --- | --- |
| TFCP2 | TNIP2 | CLIC3 | CERS4 | PPP1CB | TRIT1 | TTLL1 | LCMT1 | VPS37C | C7orf60 | ADAM8 | TF |
| MYO1C | ERMP1 | PROM2 | MAP3K1 | CYP26B1 | KANSL2 | ASAH1 | CHD4 | KIAA0895L | FCGBP | CFHR2 | IGSF3 |
| NOTCH1 | TMEM61 | ISYNA1 | C2 | CCDC102A | SGSH | LUZP1 | EVC2 | DSTN | VTCN1 | CXCL5 | SLC1A5 |
| DYNC1I2 | ETV5 | SF3B4 | CNTROB | USB1 | CCT2 | ALOX5AP | ZNF185 | PHC2 | BCL10 | EHHADH | FAM214B |
| PIF1 | EVA1B | KCNK6 | RASAL1 | APPL2 | PURB | LEPROTL1 | TMSB4X | CFL1 | HES4 | FJX1 | ABCC6 |
| HYAL1 | NR2C2AP | LOXL2 | BUD31 | INSIG1 | PTMA | DAB2 | ALDH3B1 | HNRNPD | STK11IP | PLEKHA2 | CTTNBP2NL |
| FDCSP | FOLR1 | SEC14L6 | MTFR2 | SMS | TTYH3 | LIPC | FKBP15 | HSPA12A | SLC10A1 | SOX4 | SLC10A3 |
| HJURP | MYRF | PTDSS2 | GCA | ZNF432 | HRSP12 | RERE | UGT2B10 | ENDOD1 | DGKZ | F9 | ANKRD13B |
| CENPL | ZNF563 | SC5D | UBE2M | C1orf115 | IGF1R | TM6SF2 | PLAU | ADAMTS9 | CD97 | S100A6 | APOC2 |
| NCAPG | DGAT2 | SRPK2 | HNRNPA2B1 | CYP8B1 | RFC2 | KCNH3 | MFF | GATAD2A | NUP62 | KHDRBS1 | PKLR |
| PTPN6 | TAF6 | ACSM5 | ZC3HAV1L | KIAA0930 | SIPA1L3 | AP1G2 | C15orf39 | DRAM2 | FMNL2 | TPGS2 | ELOVL7 |
| FAM109A | IL4I1 | NOTCH2 | MLLT6 | PPP2R4 | C11orf80 | SEPT9 | RAD54L | ANLN | KRT19 | DNM1L | MBOAT7 |
| PABPC4 | UNC45A | NARS | SPPL3 | TRIM28 | FTSJ1 | DBF4 | FAM49B | TNPO2 | SERTAD3 | PTAFR | SLC2A1 |
| TMEM185B | KLKB1 | NSF | HIP1R | HAVCR2 | DHTKD1 | SERPINA6 | TRIM65 | VMA21 | DYRK2 | RALA | FUT4 |
| CAV2 | CNKSR1 | SIX2 | UBAP2 | UPF3B | KIRREL | TACC3 | EEF1E1 | ACADSB | S100A2 | TTC38 | SLC37A1 |
| FAM127C | KRBA1 | STK32C | PLBD1 | SLC22A7 | KCTD9 | ACIN1 | PDE5A | PDGFRL | PTGS1 | SMARCA4 | G6PD |
| ZNF629 | CDK2 | GFPT2 | IST1 | FUT3 | CPS1 | MOB1A | TAX1BP3 | ZNF142 | CSNK1E | C16orf74 | NXPH4 |
| LPAR1 | LRP10 | BIRC7 | CD2BP2 | CCDC137 | COPS7B | PLK1 | RAB10 | ABCC5 | FA2H | BACE2 | IFT140 |
| SNAP25 | NF2 | GRN | HK2 | MAPK7 | AGMAT | MTA3 | PRPF4 | PRSS12 | SPAG1 | EPS15L1 | STX3 |
| MSH2 | PCK1 | F3 | HNRNPH3 | HNRNPUL1 | SERPINF1 | FOSL2 | MMP10 | ZDHHC1 | FLNA | PHTF2 | CERS5 |
| HNRNPM | GCNT1 | IL27RA | GLIPR2 | CHST2 | SBNO2 | MYO19 | NDE1 | SYK | CSRNP2 | H2AFY | ST6GALNAC4 |
| SUSD1 | SLC9B2 | TSPAN14 | IFI16 | HSD17B8 | TMEM56 | WHSC1 | OSCAR | FAIM | SHCBP1 | ABHD17C | DENND6B |
| SLC30A10 | JAK2 | PIP4K2C | NAA35 | ANXA2 | RBP7 | PISD | RAB31 | EFNB1 | FGFR1 | PROM1 | VEPH1 |
| ZMYND19 | CS | LRFN1 | KBTBD2 | E2F5 | RUVBL1 | SYCE1L | MCRS1 | LAMP5 | CCDC6 | SLC13A5 | TRIP10 |
| SLC26A1 | UBA1 | RBPMS | H2AFZ | DNAJC5 | DCAF16 | CHD3 | HOXB9 | MLK4 | G6PC | ATP1A1 | GAS2L3 |
| GOLGA7B | PAN3 | GSTP1 | ABHD6 | MYCBP | SLC25A19 | BAZ2A | NRM | PRKX | AGAP1 | SLC25A42 | TMEM65 |
| RBBP7 | CD276 | LRRC41 | VLDLR | RBMX | FAM219A | DOK3 | ZBED5 | HNRNPL | TRIP13 | DSG2 | MB21D1 |
| CFHR1 | TCERG1 | MASTL | PAFAH1B3 | INTS9 | PIP4K2A | CAMSAP1 | DCLRE1B | FETUB | GTF3C2 | RUSC1 | CCDC109B |
| YJEFN3 | TREM2 | FPR1 | APOBEC3C | UGT1A10 | ARSI | XPO6 | ABCF2 | YWHAH | HOXB6 | TNFRSF21 | PAPLN |
| ARHGEF1 | TNC | MYO6 | MLKL | WDR45B | MAML2 | TAT | SIN3B | TANC1 | HOXB5 | PML | SLC52A2 |
| ATRAID | TFPI2 | PPIC | SLC39A13 | HMGXB3 | RNF44 | ADHFE1 | E2F3 | FMO5 | SIRT6 | IFT80 | RCOR2 |
| CLTA | PCBD1 | PPP2R5B | DGUOK | GCDH | VTN | CENPA | CDC42BPG | TEX10 | SH2B2 | SLC4A11 | LGALS9 |
| CDKN2D | PAM | INPP5E | FHL3 | RAB3IP | RALY | TST | PRMT1 | DR1 | CD63 | CDH17 | GYS1 |
| PLK3 | CIRH1A | USP39 | PSRC1 | GAREM | RAB9A | ZNF239 | TSC22D4 | MSMO1 | FBLIM1 | SUGCT | GLTP |
| EHMT2 | MAU2 | DCUN1D5 | CASP4 | ISY1 | SYNGR2 | CYB561D1 | VEZT | UTP11L | SLC25A24 | APOC4-APOC2 | PRKRA |
| ARL4C | RORC | PCGF3 | ADAMTS12 | SNRPA | MAD2L1 | TBC1D30 | PLSCR1 | OFD1 | CNOT6 | FOCAD | ALDH2 |
| UBXN11 | NCOR2 | TBCB | DUOX1 | TMEM72 | CEP170 | FBXW9 | ASAP1 | SNAPC1 | YBX3 | CHAF1B | CHMP4B |
| ALDH1L1 | DAPK3 | MARVELD1 | FNBP1L | AQP11 | GNB1 | C15orf52 | DYNC1H1 | TMEM234 | SLC6A8 | PLXNA3 | CD47 |
| ME2 | KDM1A | GUCY1B3 | CBX1 | CENPF | ESPN | ZRSR2 | PALMD | MCU | MMP1 | GYG1 | ANKLE2 |
| BLVRA | ERP27 | GDI1 | SGOL2 | ARHGEF25 | CCAR2 | GOLGA3 | DMBT1 | B3GNT9 | TUBB6 | FUT8 | SPA17 |
| UQCRH | ZNF608 | ATP2C1 | ZNF296 | ZNF117 | BAAT | CD3EAP | SUPT5H | RHNO1 | MFI2 | TTF1 | DCXR |
| UBE2S | MASP1 | GMCL1 | ALB | AMD1 | RHBDF1 | FAR1 | PPP1R9B | DYRK3 | RBP5 | SCTR | KNOP1 |
| SART3 | GMPS | RGS19 | SLC44A2 | SHKBP1 | GLIS2 | COL8A2 | TSSC1 | B4GALT5 | POLR3D | FTCD | TMEM55A |
| KCNQ1 | WIPF2 | CCDC97 | PCGF2 | MAB21L2 | XRCC1 | E2F2 | SS18 | C9orf89 | YWHAZ | PIWIL4 | DMPK |
| CCNG2 | KIF4A | ANAPC7 | ARHGEF2 | TC2N | ADCY3 | SOAT1 | DROSHA | MYOF | HP | SOGA1 | MICAL1 |
| RGS3 | ACKR2 | SCAMP4 | ADAP2 | BRI3BP | CALM2 | CTTN | EHBP1L1 | TRA2A | MFSD6 | NBEAL2 | ECT2 |
| SFT2D2 | ADAM12 | KIAA0101 | PECR | PPFIBP1 | GRSF1 | PGS1 | PAQR5 | SRRT | EZR | BTBD10 | MARK2 |
| SH3GL1 | ACTL6A | SLC35E1 | CHN2 | SAE1 | ATF7 | ANKRD27 | TPM3 | PHF19 | GALNT7 | KIF23 | SCRN1 |
| C17orf53 | TCN1 | HLA-DQB2 | UBA2 | CSGALNACT1 | CXXC1 | TMPRSS6 | TBXAS1 | CNTRL | DSTYK | KIAA0226 | APLP1 |
| GPER1 | CDCA4 | GNMT | SLC2A6 | GPD1L | HSBP1 | MED27 | GPRC5B | GLS | SPECC1 | SH2D3A | SPATS2 |
| KPNA2 | DIO1 | MESDC1 | IL27 | ITIH2 | FAM110A | ZNF438 | CLSPN | TAGLN2 | ATP1B3 | SERPINA11 | AAK1 |
| PLEKHO1 | CDC42SE2 | FMNL3 | CCL15 | AGER | AKR1C4 | ARPC5L | RHOQ | MAP1S | PMAIP1 | STX18 | CDK20 |
| ARMCX1 | CSTF2 | ACSL1 | F5 | CTSE | RYK | IL20RA | TMEM216 | CLCF1 | BCAT1 | AP3M2 | DMGDH |
| LRRC3 | SLC51A | SLC12A2 | GYS2 | CFHR4 | ACOT12 | BARD1 | CD207 | CAPZA1 | KLF15 | FAM171A2 | C11orf84 |
| KIFC3 | GTF2IRD1 | PDPR | ACY1 | SLC7A8 | TM4SF1 | GNA15 | YWHAB | RPS6KA4 | B3GALNT1 | IVNS1ABP | LRRC56 |
| PCTP | GPRC5A | MINK1 | KLHL30 | DKC1 | PROSER1 | ITGA2 | EMILIN2 | ZMIZ2 | CDH6 | PPP1R15A | ARNTL2 |
| GFPT1 | UBE2E1 | TDG | TMEM220 | INPPL1 | KCTD2 | ADI1 | ITGB6 | RHPN1 | ADH1B | GALNT10 | SFT2D1 |
| MDFI | P2RY11 | PIEZO1 | CLIP3 | TULP3 | ZNF292 | GOLM1 | CYP2J2 | CANT1 | SEPN1 | GNPDA1 | WDR54 |
| USP21 | HPN | ARMCX6 | AHDC1 | TFF2 | CCND3 | CLCN2 | EPS8L1 | CROCC | HID1 | TMEM86A | GPRIN2 |
| CHCHD3 | MISP | SKA1 | TRAK2 | RASA3 | PTTG1IP | ARAP2 | DIRC2 | ACTB | SPIRE1 | PSMC3IP | UBE2I |
| SUDS3 | CD2AP | FAM127B | ZNF865 | SLC22A15 | ANXA5 | WDFY3 | ZBTB4 | NUP188 | TRAFD1 | SLCO1B1 | IFT172 |
| CCNB1 | EMP3 | RPP25 | PSMD14 | CMTM3 | MLF1 | HDAC2 | ADAM17 | C1orf116 | OTC | CMTM4 | WNT7B |
| ADNP2 | GAR1 | CEACAM7 | POGK | JAG1 | SMARCA5 | GPRIN1 | MAP3K10 | LRP12 | SERPINA4 | TMEM87A | C15orf65 |
| NUF2 | FCGR2A | CDK4 | SEC14L2 | KIF20A | TMEM176B | MICALL1 | RNF4 | PFDN1 | SH3GLB2 | TMEM206 | NADK2 |
| EME1 | COLCA2 | AP1B1 | CHST3 | TRAK1 | PPP1R9A | APOF | WDR62 | XYLB | WHSC1L1 | CDCP1 | DNAJC10 |
| ADAM15 | MVP | HNRNPU | GLTPD2 | GLYAT | WWTR1 | TUBA1C | TEAD4 | PYGB | ARG1 | SLC4A3 | ARHGAP1 |
| SLC39A1 | SLCO2B1 | ANKS6 | JRKL | UNC119 | B4GALT4 | SLC41A1 | STRN4 | FOXJ3 | PARP8 | TLDC1 | TGFA |
| GPLD1 | TMEM246 | CDC6 | IL15RA | MAEA | NRGN | SRPX2 | PHYH | PAK4 | CES2 | FUNDC1 | RASGEF1A |
| AGFG1 | C2CD5 | EML2 | EZH2 | IARS | B3GNT3 | AFTPH | SEMA4D | AGAP2-AS1 | KIF2C | HPD | VPS37B |
| FBXO5 | ABCA6 | CCT8 | TNNT1 | HNRNPUL2 | CNPY4 | CENPO | NFKBIE | PTP4A2 | DUOX2 | SPEG | TPM4 |
| KLF7 | PRRC2A | CKS2 | WDHD1 | HNF1B | NAP1L1 | ANXA4 | CAT | A1CF | HPSE | TNFAIP8 | PPP1R13L |
| CHPT1 | TESC | OTUD1 | SLC25A20 | CHAD | PRPF38B | SET | NBL1 | RAB38 | LDHD | LYPD6B | NDRG3 |
| SPTSSB | CDC42 | FANCI | FABP1 | HSD17B4 | GPT | ARPC1B | KLHL29 | TGFB2 | ECHS1 | DCTN2 | MSI2 |
| KIAA0907 | GARS | MARS | MELK | AKT3 | MFSD5 | GTF3C1 | PPM1G | RIC8B | PTHLH | RIN1 | ZNF532 |
| RASEF | CAND1 | TRMU | PPP1R14B | HGD | VOPP1 | PRKRIP1 | C19orf40 | NCK2 | ARHGAP27 | NCAPD2 | TSPAN15 |
| ATG4D | GTPBP2 | PPP1R12A | PPAP2C | POFUT2 | NRARP | AP2M1 | ABL1 | NCAPH | CAMSAP2 | PRSS22 | KIAA0753 |
| PLAGL1 | YBX1 | RTCA | GNB4 | SSRP1 | RABEP2 | CCDC93 | CMTM7 | CXCL6 | CNNM4 | RBPJ | LIF |
| SP1 | KCNMB4 | TNFRSF10C | RHOG | PAQR4 | IGSF23 | DMWD | CXCL3 | ARSJ | FAM222B | RAB42 | COA1 |
| UCK2 | GRHPR | ZPLD1 | TFEB | CTNND2 | TTPA | CARD11 | PRDM4 | ETNK2 | MTF2 | MOB3A | DBN1 |
| CCAR1 | ABLIM2 | R3HDM4 | FANCG | PEMT | KIAA0020 | PKMYT1 | RALGDS | RUNX1 | TRPM2 | ALOX5 | SH3GLB1 |
| GUCY1A3 | SPIB | HMGA1 | DLGAP5 | TLDC2 | SMIM10 | PACS2 | WSB1 | PLP2 | B9D2 | C4orf46 | CYP2D6 |
| ARL6IP6 | DHRS4 | CRMP1 | HTRA3 | TRRAP | MCM3 | CTDSPL | KCTD17 | INTS8 | LIPH | APOM | SH3RF1 |
| FAM118A | FCGR1A | SSBP4 | CDC42SE1 | MARCKSL1 | ALDH8A1 | HAGH | OVOL2 | TCF3 | SLC25A51 | MAPRE1 | GINS3 |
| RAC2 | C5orf28 | CCDC84 | UGT2B4 | CAD | KTI12 | KIF18B | YARS2 | AGTRAP | TFAP2A | POP1 | FHOD1 |
| NCOA5 | HSPA14 | WIPF3 | PHLDA2 | ITGA5 | NCKAP5L | QSOX1 | FUT11 | SLC36A1 | CUEDC1 | NT5C3A | SLC47A1 |
| MAP4K3 | KCNK5 | NDC1 | RALB | TRAF2 | GNL2 | ABCG5 | CASP6 | ILF3 | SLC18B1 | SLC34A2 | ECI2 |
| STIP1 | SLC24A3 | COPG1 | TMEM50A | IER3 | BUB1B | FRAS1 | DENND2D | SSPN | SZRD1 | AMBP | ADAM28 |
| A4GALT | CAPZB | SDC2 | GAL3ST1 | OLFM1 | DTX3 | RAB36 | ACTG1 | PPT1 | POR | LHFPL2 | UPB1 |
| MRPL47 | CAV1 | FAM122B | ORM2 | NRSN2 | ZCCHC17 | CDCA2 | UBE2L3 | ORC6 | RAC1 | RHBDF2 | ZG16B |
| MRC2 | FKBP10 | VPS45 | LHPP | KCNJ16 | NPAS2 | EXOC1 | KLF5 | FAM160B2 | ARPC2 | ITGAV | RTP3 |
| PLEKHO2 | VMP1 | POSTN | RCC1 | ARHGEF39 | CSNK1D | TGFBRAP1 | PIGX | MITD1 | ICMT | CBX3 | PDLIM7 |
| PUS7 | CENPM | TWSG1 | BPHL | MED25 | NUP107 | RBL1 | GATS | TRAF5 | TRIM47 | MST1R | SLC39A6 |
| CCT5 | BNIP3L | BRE | GYPC | PRPF38A | TMEM54 | MMP7 | TPD52L2 | EPN3 | IFNGR2 | ADH4 | KIAA1524 |
| SLCO3A1 | ATXN2L | AKAP8L | NUFIP2 | VEZF1 | KLHL42 | MAST2 | FAM64A | FAM60A | OSBPL3 | MANBA | FZD7 |
| CFTR | OXTR | RIBC2 | ABCG8 | C9orf116 | GTPBP4 | RAP1GAP2 | PRR11 | PXN | NACC1 | OSBPL5 | SMIM22 |
| WDR1 | GRAMD1A | OLA1 | SH3BP1 | PLEKHM2 | WDR75 | HN1 | XPO7 | CIZ1 | DOK1 | FGD6 | PDP1 |
| TROAP | TSC1 | TMED3 | BUB1 | FBRS | WNK2 | TIMP2 | ASGR2 | AGR2 | PPP5C | PLIN3 | SLC6A12 |
| PHIP | FAM220A | BRIX1 | ABCC10 | PON3 | ZC2HC1A | APOE | ZMYND8 | ITGAM | SFPQ | TMEM237 | GBGT1 |
| S100B | STK38 | ASNS | ALDH7A1 | DDX23 | PPP1R14C | RBM14 | PLEKHG2 | WDR34 | CYP4A22 | CTDNEP1 | WDR91 |
| MFAP2 | HS3ST3B1 | QPRT | RACGAP1 | MARCKS | NECAP2 | YWHAQ | UPP1 | MIDN | ATP6V1F | GPR56 | BTF3L4 |
| SAR1B | C2CD4A | CYBA | CHST1 | OPN1SW | RQCD1 | BZW2 | SMKR1 | ELOVL1 | RFWD3 | STX6 | NCBP2 |
| INCENP | TANGO2 | EPHB3 | PQLC1 | NEBL | HECTD3 | ZBTB17 | TSEN54 | TMEM87B | ABCB4 | SLC7A1 | DNM1 |
| ENPP5 | TIMM22 | MCM4 | KIF2A | TES | TMEM147 | TSPO | FOXK1 | AGMO | PIAS3 | HMGCS2 | NUP93 |
| CCNB2 | PPAPDC1B | NUP43 | DBR1 | STK17B | CDCA8 | TESK1 | DDX11 | PDCL | CSF1 | GAL3ST4 | TOR4A |
| STK36 | TMEM156 | NELFB | C4BPA | SMURF2 | CCDC9 | AIG1 | E2F6 | FUNDC2 | RRAGC | CXCL1 | KRT80 |
| GNB2 | PJA1 | BTD | SRSF3 | PLXDC1 | FOXK2 | ARMCX2 | TP53BP1 | ALDH5A1 | CDK19 | PKP3 | ODF2 |
| ARPC3 | ADORA3 | MMD | TCOF1 | WASF2 | AEBP2 | MRE11A | RGS1 | ZSWIM6 | CAP1 | PFKFB1 | TBC1D22B |
| H2AFX | EPHX2 | HOXB13 | CCL26 | GBAS | HCFC1 | SH3PXD2B | PELI1 | PTPN2 | MECOM | SPHK1 | GLYATL1 |
| DCTN1 | MARC2 | SEMA4A | SEMA3C | FBXO18 | OBSCN | STRIP2 | MBOAT4 | OSBPL7 | AADAC | CA5A | RNPS1 |
| CDK2AP1 | CYP2C9 | USP1 | UBE2J2 | RAD51 | FGA | ARRDC1 | SORD | ITGB1BP1 | ZNF195 | CDH24 | TMC5 |
| NR0B1 | HNRNPDL | QSER1 | HAUS6 | SDC3 | MKI67 | GAS2L1 | METRNL | ALDH6A1 | SLC38A4 | STARD3NL | FUOM |
| SLC43A2 | ZNF146 | ADCY5 | SF3A3 | ACSS1 | GPN1 | SIGLEC10 | C8A | GTSE1 | RAI1 | RAVER2 | ANXA3 |
| MSANTD4 | NEDD1 | OSGIN1 | ADPGK | KDM5C | SETD8 | PXMP2 | C4orf48 | ADO | CREB3L1 | KLHL17 | EXOC6B |
| IL13RA2 | PARD6B | GAMT | ITPR2 | CCDC14 | PRR12 | EFTUD2 | EWSR1 | ANKIB1 | CYP4A11 | HIF1A | EGLN3 |
| PMP22 | FZD6 | CNTNAP1 | PLEKHS1 | ZNF486 | CSPP1 | PTPN13 | CDCA7L | KIAA1244 | CPSF6 | NRF1 | LAMA5 |
| DDX27 | MTPN | PSMD2 | PLK4 | PPP2R1A | ANKRD11 | CHST4 | C21orf59 | FGD3 | CBX5 | FAM102B | C6orf132 |
| SLC7A9 | NUTF2 | PYGO2 | SMTN | TPM2 | PFN2 | KIF18A | C8B | CTHRC1 | MRPL17 | MKL1 | PPME1 |
| MEX3C | CSNK2A1 | ZNF664 | BHMT | ZER1 | MTHFD1L | PRODH2 | CDHR5 | CHPF2 | RNF34 | RARG | PNMA1 |
| ENOPH1 | KCNJ8 | POU5F1 | MTX2 | FUS | RBBP4 | RBM38 | ZMYM4 | DLGAP4 | SH3BGRL3 | RASSF6 | RBBP8 |
| NABP2 | DTNBP1 | CDC20 | PI4KB | SNRNP40 | RAN | SHMT1 | VCL | NEO1 | C20orf194 | MBOAT2 | AFM |
| WDR77 | MCM10 | TM4SF5 | HRG | MED10 | DPCD | B4GALNT3 | SAA4 | SYDE1 | CTSC | STK17A | C11orf49 |
| GNG5 | TTC22 | COL18A1 | HEATR6 | CYTH3 | FXYD5 | TGFB1 | MAPK13 | CXorf38 | DUOXA2 | NPEPPS | ABAT |
| EXO1 | ENO1 | BAIAP2L2 | SOCS7 | ALDOB | APOBR | SMLR1 | MCOLN3 | ZNF207 | ME3 | IFFO2 | RASSF9 |
| MCM6 | GRAMD1B | SRI | SNRPD1 | CCDC24 | FAM105A | DEAF1 | GTF3C4 | CENPE | PMEPA1 | SLC27A2 | RBM17 |
| NOV | CERS6 | CDCA3 | AFAP1 | RPRD1A | FUBP1 | IFT52 | KIF11 | CLSTN1 | PHLDB1 | CERCAM | TMEM184B |
| FKBP1A | CDYL | KDELC2 | SMOX | RDH16 | S100A3 | GPAM | ATP13A1 | SEMA3E | CRABP2 | PC | HAGHL |
| FAM89B | HMGN1 | FCHSD1 | DPH7 | PF4V1 | MAX | ACOT9 | AHNAK2 | GAREML | CBL | LAT2 | BCAR1 |
| ARHGAP18 | LUC7L3 | TWIST1 | C17orf67 | UBALD2 | SCD5 | LUC7L | TMEM201 | C2CD2 | STIL | BICC1 | RUSC2 |
| RFC4 | NDOR1 | JOSD1 | CD302 | PLD2 | CDC7 | SAFB2 | DTX2 | BHMT2 | ARHGAP39 | SLC9A7 | CLIP2 |
| MGMT | PRKAR2B | PTPN12 | WARS2 | DHX15 | CORO1C | E2F4 | ORC1 | PDE4A | DPYS | SPINT1 | CTXN1 |
| VASH1 | LINC00998 | ASF1B | MTHFD2 | FOXF2 | POF1B | CLIC1 | COL16A1 | SELENBP1 | NAGK | NCS1 | HNF4A |
| MAST4 | LGALS1 | ENAH | FANCD2 | UNC93B1 | USP33 | GALNT3 | TRIM32 | C2CD4C | CPN1 | SAMD4B | C3orf80 |
| NASP | PPM1H | KIAA0319L | FAM213B | GLUD1 | ANKRD13C | IL18 | FAM65A | CALU | GLG1 | C12orf49 | CYP4F2 |
| RBX1 | AP1S2 | SNN | ARID3C | ORC2 | DSEL | C19orf54 | SMYD5 | TMSB10 | LRFN4 | GPSM2 | TANC2 |
| ICAM1 | BBX | SLC17A2 | FANCL | LRRC1 | ZNF121 | KLC3 | TCTEX1D2 | ACTR3 | KIAA1522 | TMEM43 | LPCAT1 |
| PYCARD | TLE3 | TNFSF13 | VRK2 | MADD | CHEK1 | IQCB1 | PNKP | POLD3 | GULP1 | EFHC1 | TTR |
| DEPDC1 | SUGP2 | WTAP | RAB28 | DNMT1 | CHTOP | NUP205 | FAM155B | RAP1GAP | CABIN1 | ZNF618 | GJB3 |
| SEPT7 | SMPDL3B | MTTP | UBAP2L | PTPLA | CBX2 | NIPA1 | RAB35 | CNOT3 | F10 | LARP4B | AZGP1 |
| C1QTNF1 | ERO1L | STX2 | CD300A | BRD9 | KCTD11 | C14orf132 | F11 | TNFAIP2 | PAFAH1B2 | FAM98B | DRAM1 |
| JADE2 | NELFA | APH1B | CDC45 | QDPR | MAPK15 | SLC6A1 | RNF144A | PKD2 | HIATL1 | GSK3A | IKBKE |
| SPI1 | FGG | MOB3B | CEP95 | FMNL1 | GSTA1 | TP53I11 | DEPDC7 | PRKCI | CYTH2 | HAAO | CHST11 |
| LMO4 | PQLC3 | TONSL | HDAC1 | RAP2B | SLC4A7 | PRKD2 | UCHL1 | TRAPPC4 | SUPT7L | TMEM136 | CREB3 |
| NCAPG2 | MGST2 | DVL3 | PTGES | ADORA1 | NAP1L4 | GJB1 | TUBG2 | DNAL4 | KPNB1 | C9orf172 | MPP6 |
| ACAA1 | FRMD4A | PAPSS1 | COPG2 | CDC25B | SEMA4B | GPC4 | PANK1 | RAVER1 | PDE7A | GPR107 | OSMR |
| HOXB7 | COTL1 | CA11 | LRRN2 | TUBA1A | PI3 | SERPINB6 | BICD2 | ANKRD52 | IER5L | LDLRAD3 | KCTD10 |
| ZNF512 | LDOC1L | SRSF2 | SLC25A15 | FOXJ1 | XRCC2 | FAM102A | MZF1 | SRCAP | SERPINH1 | TSPAN3 | STX7 |
| ZNF160 | TNFRSF18 | UBE2D1 | ZNF580 | IFI27L2 | CAPNS1 | RNASE4 | U2AF2 | FAH | CBFB | XXYLT1 | ACYP1 |
| MYBL2 | UHRF1BP1 | SLC39A10 | XRCC5 | ZNF385A | POLA1 | NSMAF | RAB11A | PPIH | DNM2 | PTBP3 | DAO |
| C7orf31 | VANGL2 | NDC80 | ANKS1A | AMZ2 | CCT6A | CCNJL | NAGS | FSCN1 | FRMD8 | ADPRHL2 | TNIK |
| MYO1E | FAXDC2 | OSGIN2 | KIF5B | FBXO46 | COL11A1 | PPP1R14D | C1orf216 | NCKAP1 | THRA | STX1A | C3orf52 |
| C1S | C19orf33 | TUBA4A | CYCS | RNF19A | DHRS12 | S100A8 | CSF3R | CD14 | ASRGL1 | PFKP | CDO1 |
| PPP1R3G | HLA-DMB | FERMT3 | SHC1 | SELO | F2RL1 | GALT | PDK3 | HMGCL | DHX37 | TBC1D10B | ITGA3 |
| SMIM3 | RABAC1 | SRM | PRSS21 | PPM1M | SOCS1 | SELM | BSG | CCL15-CCL14 | DMTF1 | GPD2 | ITPR3 |
| TBX3 | MYRIP | ECHDC3 | DDC | SUCO | PRR7 | FNBP4 | RCE1 | NEXN | GALNT18 | SERPINA10 | SERPINC1 |
| ADD3 | NUGGC | ARHGDIB | FAM210B | PEX11A | ALDH4A1 | CHMP1A | EIF2S3 | NCF2 | TACSTD2 | RNF145 | PKM |
| NCOA7 | SLC22A3 | COQ10A | KMT2E | KDM3B | HEG1 | MSC | IL2RG | PARVA | ATIC | PRAP1 | ANO9 |
| DSN1 | SAMD5 | PAG1 | FGD1 | VEGFB | GPR160 | ADAP1 | SERPINE2 | PITRM1 | UNC5B | ASB6 | F12 |
| IL18BP | TSPAN4 | CMBL | TNFRSF12A | LYPD1 | GPR176 | VEGFA | GAS7 | PRKCD | ACVR1B | ARF3 | PIPOX |
| UCP2 | EVC | SAMD11 | PIM3 | CKAP4 | TTC39C | TMEM194A | UBE2T | IBSP | SPIN1 | ANKRD13D | PLCD3 |
| CDS1 | BCKDHB | PYGL | LCAT | SRRM3 | SLC30A1 | PRSS16 | TOMM34 | BLMH | ARPC1A | METTL9 | NCDN |
| ALDH18A1 | ATP7B | GLB1L | ITIH4 | TK1 | CAPN1 | ZDHHC2 | IP6K2 | FLII | KANSL1L | PLAUR | LIMK1 |
| GAST | LRP11 | MMP11 | PXDN | TFRC | IYD | GAPDH | PLEKHG4 | ACVR1 | CYB561 | MGAT5 | ABCC1 |
| LY96 | C1orf162 | COL4A2 | GPI | SERPINA1 | SOCS3 | FLOT1 | EHF | TBC1D8 | OSBP2 | TMEM159 | LRP8 |
| MPST | DSG1 | ELL2 | SNRNP200 | SLC43A1 | BCL2A1 | DAGLA | NOP58 | PLEC | LLGL1 | SLC38A1 | G6PC3 |
| LPHN1 | PKN1 | RPS6KA1 | LSM1 | ETFB | CBX6 | MAMLD1 | CYS1 | MBNL1 | CHTF18 | HAO1 | LIMCH1 |
| NPTXR | WASF1 | PBLD | ECHDC2 | KCNJ11 | CYP2B6 | C1orf106 | BIRC5 | SNTB1 | TMEM69 | SLC6A6 | SLC37A3 |
| STS | TMEM45A | COL12A1 | PEX11G | SAMD9L | PLEKHB1 | PBK | ILF2 | HNRNPA1 | ACTN4 | PIP5K1C | KIF3C |
| ST3GAL4 | DNASE1L3 | ATF4 | FAM20A | MOCS1 | PDGFD | AKR1B1 | RIN2 | ARF5 | ACD | APOC1 | LPCAT4 |
| MDFIC | SYNJ2 | CX3CL1 | ZBTB47 | FAM127A | MYO1F | DFNA5 | ATXN7L3 | FBP1 | SRC | EPHB6 | RGN |
| GLOD5 | WWC1 | PPP6R1 | ZNF880 | PSENEN | FEN1 | ACOT2 | TBKBP1 | RELB | HLF | HILPDA | MLLT3 |
| DDX39B | TMCO3 | SOWAHB | ZC3H12A | SFXN5 | COL1A1 | MCM8 | CDKN2B | ORMDL3 | SF3B3 | ACSM2A | APOC3 |
| UGT1A1 | CBR3 | FAM101A | HP1BP3 | PFDN4 | GCHFR | ABCC2 | MCAM | MUC6 | ZBTB12 | HDAC7 | C12orf75 |
| SPG20 | CHST13 | CORO2A | WIPI1 | ABCA3 | VCAN | AR | SMPD4 | SUMO2 | RRAS | SYT13 | ORAI2 |
| HIST3H2A | GPR183 | CELSR1 | RAPGEF1 | SMOC1 | CCL28 | RENBP | CLASRP | ACAT1 | SNCAIP | RBMS2 | SLC9A1 |
| ETV7 | ADAMTS10 | LIMD2 | C10orf32 | TAF1D | USH1C | DENND4B | DYNLT1 | LIMS1 | WSB2 | AGXT | ARMC9 |
| CD53 | XPOT | DHX32 | CASK | RAB3D | CLDN10 | HN1L | ARPC4 | SAMD9 | GTF3C6 | ODF2L | MSANTD3 |
| FERMT1 | TRAPPC1 | DYNLRB1 | PDCD5 | HNMT | PDGFC | FNDC3B | GCSH | CDCA5 | KRT17 | MYLIP | RBP4 |
| CARD10 | C3AR1 | ACAT2 | SLC5A1 | PCED1B | PKIB | TEAD3 | PPRC1 | DMKN | ENTPD4 | ADAM9 | FHDC1 |
| CHST9 | ACLY | WARS | TYROBP | ZWINT | JAG2 | SLC5A3 | ZNF579 | ARHGEF5 | POLD1 | MLXIPL | AHSG |
| SPC24 | CENPQ | PIP5K1A | EXT1 | RERG | THSD4 | SLC2A9 | DHRS3 | GNS | TPX2 | PGM2L1 | MASP2 |
| ELFN1 | ABI3 | ODC1 | TRPV2 | ZDHHC20 | HRAS | CD59 | EDA | COMP | CIC | ANG | CDR2L |
| NEDD4 | SNAI1 | C10orf35 | TCEA3 | BTNL9 | KAAG1 | CSNK1G2 | TLR1 | FAM189B | CYP7A1 | TNFRSF11A | MST1 |
| C19orf48 | COL6A2 | MICA | DEF6 | PRRC2C | CTH | TTK | VPS41 | SERPING1 | FOXM1 | ETV6 | KNG1 |
| ANKRD22 | CD37 | PLTP | RIPPLY1 | KEL | PAOX | PLXNB3 | HOOK2 | HAUS1 | KIF14 | DDA1 | RABL6 |
| GPX3 | NDUFS5 | KDELR3 | PRKDC | LASP1 | MX2 | S100A4 | C20orf96 | XYLT2 | SEC61A1 | TFR2 | TMEM132A |
| ENPP1 | GPD1 | FAM50A | SKAP2 | MROH1 | MYO10 | RAB20 | SPATA20 | ASL | JAK3 | LRRC8B | CYP27A1 |
| BAMBI | GCAT | MEX3D | STAB1 | MAFG | TLN1 | CTTNBP2 | TRNP1 | ZRANB2 | MILR1 | LMNB2 | SERPIND1 |
| GGH | PLEKHH3 | CCNI | HGF | EIF3B | LILRB4 | FH | TUFT1 | RRAD | IQGAP2 | PAH | S100A11 |
| SUSD4 | HLA-DQA2 | SERPINA5 | GLT1D1 | DTYMK | PIK3CD | GMNN | NXT1 | CAPG | FAM53B | PLCB3 | CPN2 |
| PACSIN2 | RETSAT | SLC35F6 | CYP2E1 | POLR2A | ADSS | MYL6B | ESRP1 | SPTLC1 | PROZ | GLYCTK | TTC39A |
| ST3GAL6 | PLEKHA4 | WDR72 | KITLG | KIAA0040 | DNMT3A | STMN1 | FAM208B | CCNF | ENPP7 | ITIH1 | ELF4 |
| ARL4D | DCK | PBX3 | FAM3C | S100A13 | RANBP1 | NUSAP1 | PRNP | PLAGL2 | CPNE7 | SFXN3 | PROC |
| INTS3 | COL5A2 | DDT | MXRA5 | RAB8B | ADCY6 | CD86 | ABCB11 | ARHGAP33 | TMEM102 | KCTD1 | TMEM51 |
| ETFDH | ARMCX3 | UBE2E2 | FAM129A | PVRL4 | HEXIM1 | TPI1 | CD151 | ARHGAP4 | SRSF11 | FZD2 | ACOX2 |
| CFD | SLC16A2 | SMIM6 | KLK11 | ACSL5 | CADM1 | DCAF11 | PLIN5 | TRIO | TAP1 | GMIP | HPR |
| KIAA1161 | CCNA2 | TNFAIP8L2 | EIF4G2 | KDM4A | AGBL5 | HSDL2 | NR1H4 | CCNO | LIMK2 | DBNDD2 | NCEH1 |
| RMI2 | ZNF28 | C1QTNF6 | TRIM55 | FLOT2 | UGT1A4 | ARHGEF19 | C15orf48 | ATAT1 | PRPF39 | FAM129B | SARDH |
| PRAME | SERPINB1 | LRRC42 | SNRPB | LGALS3BP | CAPN2 | COL10A1 | TUBA1B | SFI1 | KCNF1 | EPHX1 | PFKFB3 |
| MYH9 | CKLF | M6PR | ALYREF | P2RX4 | CLDN15 | MXD1 | SULF1 | PLBD2 | PA2G4 | METTL7A | MAT1A |
| CFH | SLC44A4 | DMTN | TIMP1 | PGLYRP2 | ADNP | ACOX1 | SUB1 | CDKN2A | IVD | OGFRL1 | SLC35E4 |
| ADAMDEC1 | ARRB1 | AKIRIN1 | SAMSN1 | ALAD | NCF4 | RPA1 | CHML | SRSF9 | RP11-1220K2.2 | ASPDH | CA9 |
| FYB | SHBG | PCNA | TUBG1 | ITGB5 | PLXNB2 | HBEGF | DECR2 | OCEL1 | TPPP3 | ITIH3 | TPBG |
| RFX5 | C2orf72 | CAPN6 | ALDH1A1 | FADS3 | SLC35B2 | RAD51AP1 | ENTPD2 | ENKD1 | TMEM176A | AP4M1 | RNF24 |
| TSKU | HAVCR1 | BTG3 | PPARA | LTB | SEPW1 | FOSL1 | BTBD3 | RIPK2 | PSPC1 | TMC6 | TMTC2 |
| ATP8B2 | FOXS1 | TMEM91 | BMP6 | AKR1D1 | SMC4 | GGA2 | TRAIP | ALAS1 | JMJD6 | AMPD3 | LPAR2 |
| C1orf233 | MUT | NUP210 | ZBTB41 | UBL4A | SHF | RPL17 | UNC119B | POLA2 | WIPF1 | GCNT3 | MUC1 |
| HGS | SLC7A5 | RCAN2 | TNFSF13B | ZIC2 | BBC3 | UBE2Q2 | SPDL1 | ABHD14B | SLC25A13 | ARL14 | SGPP2 |
| IFNLR1 | CST2 | ARL16 | SH3BGRL | OCRL | C11orf54 | TFE3 | PRC1 | TOPBP1 | AES | AGRN | VASP |
| TSPAN13 | KCTD15 | CEACAM1 | RASSF2 | SLC16A7 | DDOST | PTTG1 | NOP56 | YWHAG | KIFC1 | ZSWIM4 | F2 |
| EBPL | RCL1 | RBMS1 | FCER1G | ZNF692 | BAX | NAT8 | INHBC | PSMG3 | DDX39A | HSD17B6 | RAB6B |
| CITED4 | SEC61G | HCFC1R1 | CACYBP | COL6A3 | SEPHS2 | CPNE2 | PGRMC1 | CARD6 | TACC1 | MEIS2 | EFNA5 |
| FPR3 | APBB1IP | NPM1 | TNRC18 | CKAP5 | RHOC | SLC22A9 | KIF13B | TOP2A | DENR | MED22 | F7 |
| PLIN1 | FAM26F | OGT | CLIC6 | SLC39A5 | ACTR2 | THOC6 | ABCC4 | FAM131C | SWAP70 | NUMBL | MFSD10 |
| CORO1A | C5AR1 | TGIF2 | DVL2 | ADK | NEK2 | DHRS4L2 | FANCE | EIF3D | SCCPDH | PPP1R37 | SLC27A5 |
| DDIT3 | SKIL | LGALS3 | RAD21 | EFNA4 | GATM | ILDR1 | ITGAX | HMGN4 | FAM213A | PTGFRN | PCK2 |
| LTBP1 | HIP1 | FKBP4 | CCL2 | EPS8L3 | IQGAP3 | SGOL1 | IKBIP | LZTS2 | SMPD2 | IFT57 | PLG |
| SELPLG | ADRA2A | LSM7 | C9orf142 | SFRP2 | MSR1 | IGF2BP3 | VIM | SLC1A2 | LAMP3 | SMG9 | PLEKHB2 |
| HMMR | C14orf105 | SLC17A4 | COPB1 | SNRPA1 | ACY3 | SLC6A13 | MLLT1 | TIGD1 | OAF | DZIP1 | GIT1 |
| CD109 | UGT2B7 | EIF4EBP1 | AKAP7 | TARS | RNASET2 | ORM1 | PRPF3 | CFB | LAPTM5 | MTA2 | UBASH3B |
| WNT4 | TMEM163 | LST1 | RNASEH2A | RRM2 | EPRS | CXCR4 | LFNG | TNFSF9 | NUDT1 | AMDHD1 | BHLHE41 |
| RAB32 | GLRB | ZNF468 | NES | CYBB | PDXK | ENTPD5 | GRB7 | TSR1 | UGCG | MMP14 | FAM57A |
| CEBPA | B3GALT4 | ITGB2 | IGFBP2 | SCN1B | SLBP | NOP2 | ATAD3B | ARHGAP11A | DHX40 | MICALL2 | PRMT2 |
| HSP90AB1 | HSPH1 | CREB3L3 | C8G | CCNE1 | LOX | SLC12A8 | AP5Z1 | ABHD11 | ANKRD10 | YEATS2 | ZDHHC7 |
| SCP2 | PCSK6 | LOXL1 | WBP5 | OSTM1 | GSKIP | BCL9 | KLHL9 | BTN2A1 | ETNPPL | ADORA2B | OSBPL10 |
| ACADL | IRF1 | FOXC1 | COBLL1 | VWA5A | SCNM1 | WFDC2 | ZG16 | CLDN4 | HCN3 | SPP2 | TYRO3 |
| TINAGL1 | GSTM3 | EPDR1 | GPSM3 | TNFRSF11B | SLC4A2 | DCTPP1 | MCM2 | AXIN1 | TMEM60 | F13B | HFE2 |
| LRP1 | XDH | TMEM181 | ITPRIPL2 | EIF5A2 | PLOD3 | SLC22A1 | CDK1 | CCND2 | ARL2 | UBE2Z | ASGR1 |
| LAD1 | IRAK1 | SPATC1L | REEP6 | LIMA1 | CHAF1A | KIF15 | ADM | HDHD1 | STK38L | CACNB3 | C5orf30 |
| IGFBP1 | CTSV | TMPRSS3 | KLF16 | HEY1 | HSF4 | NDUFA4L2 | PTPRM | NUP85 | CAPZA2 | LAMC2 | TMEM44 |
| ATP11A | EPHA2 | RASSF7 | CGNL1 | FABP3 | MARC1 | EMB | SNRPG | RFXANK | NUDT7 | RSPH1 | SERPINF2 |
| DDIT4 | SMAD3 | TNFSF12 | NONO | MDC1 | PPARD | DEGS1 | STXBP2 | SLC44A3 | NT5C | GABBR1 | BDH1 |
| BCL2L11 | CPA2 | MAL2 | ABCA2 | ELOVL2 | CD248 | MAPK8IP3 | RRM1 | KRT10 | LSM14A | TM7SF2 | PLXNA1 |
| CYP2A7 | ATOX1 | GCGR | LCP2 | CYP4F11 | PWWP2B | HMGN3 | POLR3C | NPC1 | ACAP3 | DDR1 | SMARCD3 |
| ADCK3 | PNPO | NOL3 | EDN1 | TMEM243 | HKDC1 | CES3 | OLFML3 | RNF122 | BCL3 | DCTN5 | APOA5 |
| NTN4 | MXRA7 | GUCA2A | TYMS | MSN | C14orf1 | SEPT5 | CAPRIN1 | BLOC1S3 | RNF19B | INPP5J | RHOV |
| CA12 | B3GNT5 | DCDC2 | DNER | MAGEF1 | CCDC64B | TFF1 | FNTA | MIER2 | XPR1 | KHK | ALDOA |
| CA14 | CD83 | PRRC2B | PLD1 | ARRDC2 | SH3BGRL2 | MOGAT2 | ACBD4 | PRR13 | RGS10 | GPX8 | PACS1 |
| CYSTM1 | NPC2 | HIST1H3D | ACSS3 | UBR5 | MBD6 | PEA15 | SKA3 | NMB | TXLNA | MTMR2 | CCL16 |
| HES1 | CD82 | MPV17L | BDKRB2 | C3 | C16orf59 | YARS | DCAF7 | UBE2C | WDR3 | LARP6 | GIPR |
| CLEC11A | DOK7 | TUBB4B | HIGD1A | ANP32E | NEK6 | PTGES2 | RASSF3 | CENPW | UBE2E3 | DLG3 | SLC35F2 |
| SORL1 | GLIPR1 | DYRK1B | CDR2 | SIPA1L2 | ZIC5 | SMARCB1 | ANXA13 | MSRB3 | SAMD1 | ABI2 | IMPDH1 |
| LXN | TBX2 | IRS1 | PUSL1 | VAV1 | MAPK12 | GPATCH8 | C11orf53 | CKAP2L | IGFBP6 | AQP9 | CEP55 |
| VSIG4 | MZT2A | MAF1 | TSPAN12 | PTRF | CSF1R | GOT2 | GGCT | PPIA | CACHD1 | APOA1 | NFE2L3 |
| P4HA2 | TMEM74B | RPS2 | ANKRD1 | TGFBR3L | TUBB | PGF | POLE3 | RPA2 | SLC52A3 | LECT2 | DUSP4 |
| SORT1 | SRRM2 | RDH5 | CYTH4 | GSR | SUOX | NT5DC2 | TPRN | MBL2 | ADA | MPZL1 | A1BG |
| AIF1 | LDHB | HK3 | WDR6 | CDC42EP1 | EDIL3 | COL5A1 | CHN1 | CHMP4C | DSCC1 | FCHO1 | ABR |
| FOLH1 | GPC1 | AOX1 | TCN2 | SULF2 | ECM1 | BOK | EPOR | SPDYC | CES1 | CD58 | YKT6 |
| E2F1 | ABCA8 | GOLT1A | PNP | LEPR | IGBP1 | SMC5 | MAPK3 | PDZD11 | TSN | ITGB8 | ZDHHC13 |
| PTK7 | DHODH | HSD11B1 | RP5-966M1.6 | ARHGEF16 | PRKCDBP | RARRES2 | RECQL4 | CYP2A6 | BTBD1 | BAK1 | TMEM165 |
| EFEMP2 | RGL2 | ITPA | SPSB2 | HMGB2 | SMAGP | ACOT1 | MED12 | HAPLN3 | ZNF320 | MPV17 | HPX |
| PTP4A3 | ABCG2 | NEB | POGZ | WNK3 | UTRN | B3GNT8 | CHST10 | EPB41L2 | TMEM164 | CHMP3 | SSBP2 |
| ID4 | OSBPL9 | ZNF496 | PDLIM3 | NUAK2 | METTL1 | CUL4B | ADRB2 | ZMIZ1 | APOC4 | FAM171B | MITF |
| MYCL | MUC3A | EVI2A | GPT2 | MZT1 | COMMD8 | B4GALNT4 | PES1 | MNDA | ARHGEF17 | PTPRE | MAP3K6 |
| SURF2 | SUCLG2 | SNRPF | SH3YL1 | IL4R | NXN | ATF7IP2 | SNX12 | PFKFB4 | FABP5 | SMARCD1 | PPP2R3A |
| CUX2 | KMO | TAPBP | EPS8 | MCL1 | PFN1 | TGFBR1 | TMEM151A | GINS1 | PCYT2 | ANKS3 | NR1I3 |
| TMEM106C | USP22 | SIGMAR1 | ISLR | CCDC28B | ABCG1 | GSTO1 | MXRA8 | SLC9A9 | HTR3A | MPP3 | ZNF213 |
| FOXA2 | ASPHD1 | CKS1B | DAK | LPAR6 | IGFBP3 | KIAA0100 | PLXDC2 | C7orf49 | LAMTOR5 | AACS | ENO2 |
| PYCR1 | TMEM125 | GNA12 | C6 | TMC4 | TWF1 | VPS35 | HNRNPR | PAK1 | PVRL1 | NAA40 | OTX1 |
| C1orf122 | RTN2 | NAT14 | MEX3A | CMTM8 | MKKS | CLYBL | RAD9A | NFKB2 | FABP6 | SEPT2 | ATP13A2 |
| MMP2 | RAB11FIP1 | KCNE4 | HK1 | ASS1 | CLDN14 | ARHGDIA | ANXA11 | LDB1 | YDJC | TRIM45 | CCDC74A |
| FGL1 | CYP3A4 | TPCN1 | ISOC1 | BRMS1 | SSNA1 | PON1 | SLC17A1 | ACOT7 | ACOT4 | ADH1A | SLC16A3 |
| MTSS1 | IL6R | CENPU | APP | SLC25A47 | CLTB | PPP2R2C | LPA | ADAM10 | IFRD1 | APOB | ADH6 |
| MYEOV | SLAMF8 | SMAD7 | POLE4 | FAM150B | PDRG1 | MTHFD1 | IER5 | CDH11 | AP2B1 | TCTN2 | CPB2 |
| PM20D2 | ATP1B1 | DPYSL3 | PRTFDC1 | CCDC64 | TTC36 | SPRYD4 | PCCB | CDT1 | MMP12 | PITX1 | APOH |
| GSTA2 | MPDZ | ACAA2 | ELF3 | TOP1 | ETNK1 | RIOK1 | SCPEP1 | NFKBIZ | GC | GLIS3 | ANGPTL3 |
| CRYAB | PSIP1 | CNGA1 | CIB2 | CFHR3 | SSH3 | MGME1 | AURKB | OLFML2B | RPIA | LAMB1 | MAP7D1 |
| MBOAT1 | EHD2 | FBXO31 | COL3A1 | CFHR5 | DUSP12 | FBLN1 | PPP1CC | DHRS1 | PRAF2 | RAB11FIP5 | PEBP1 |
| ITGB1 | RRAS2 | FCGR3A | MMP9 | CFL2 | CUL7 | PKP4 | PLXND1 | MFGE8 | BAZ1B | IQGAP1 | RCC2 |
| CYP2C8 | WDR76 | ANXA1 | ELMO3 | GIPC1 | TFDP1 | OAZ1 | SEPT8 | CCDC88B | VGLL4 | TNFAIP6 | CRYL1 |
| PHPT1 | SH3KBP1 | RNASE6 | C1orf198 | CTSK | APOBEC3G | SSB | VAV3 | DHX9 | AXL | SLFN13 | RELT |
| SULT1C2 | LLGL2 | ASPH | SNX7 | IDI1 | THEMIS2 | SIRPA | RCAN3 | ANP32B | KIF12 | DEPDC1B | RIT1 |
| SLC35D1 | CDCA7 | MGST1 | GPNMB | ALDH3A2 | FXYD2 | SOX9 | SNRPD2 | AATF | ARFGAP1 | CTBP2 | SEL1L3 |
| HSD17B13 | LRRC25 | NOTUM | HSPA6 | ANKS4B | ENTPD6 | ITM2C | COL4A5 | SNRNP70 | FAM89A | CCDC43 | PIGS |
| FNDC1 | PROCR | SHD | B3GNT2 | NLRC5 | NR1I2 | ACTN1 | ST6GAL1 | KIFC2 | PCNXL3 | SEMA6A | CYP4F3 |
| CYP3A43 | GINS2 | PAQR8 | STARD10 | CLEC2B | RHOBTB2 | SMC2 | TIA1 | RBM12 | SP140L | FKBP9 | ACSM2B |
| RPS3 | CNFN | RAB34 | BSPRY | BIRC3 | CAPS | NPTX2 | TOMM40 | FMO4 | NCL | RAB27B | RASSF8 |
| GBP2 | MPPED1 | STXBP1 | NKD2 | EBP | LRRC59 | PALD1 | FMO3 | NRP1 | CBLN1 | ATN1 | IQCE |
| SHROOM1 | SOX12 | CKAP2 | SERPINB9 | RANGAP1 | GGT6 | RAB25 | FDX1 | LMCD1 | STC2 | TTF2 | FBXL19 |
| COL4A1 | KCTD12 | GOLPH3L | CD44 | SPP1 | CETN2 | TMEM82 | CNOT11 | AGXT2 | PGK1 | IQSEC2 | SPRED1 |
| CTSS | CHSY1 | WDYHV1 | SLFN11 | CLIP4 | TLR2 | LIG1 | DESI2 | LENG8 | HUNK | UAP1L1 | HOMER3 |
| THRSP | DAPK1 | TCF19 | SOD1 | PITPNM1 | DNAJC9 | LMNB1 | KAT2A | IL1RN | TRMT1 | SULT2A1 | SLC2A2 |
| MAOB | PGD | SBF1 | ZNF266 | CLK2 | EFHD2 | SFRP5 | PRSS23 | CYP4F12 | SPATS2L | ARNT2 | PHF21A |
| MYADM | MCM5 | SERPINA7 | TMEM173 | DAB2IP | LAMA4 | SF3A2 | MCM7 | KNTC1 | SLC22A25 | HDDC2 | FAM117B |
| IL34 | UNC5CL | MMAB | DTL | GAS2 | ETV1 | ACSF2 | PKDCC | POLR2H | TBL1XR1 | LEAP2 | FZD1 |
| TTLL4 | GALK1 | C19orf80 | HMGCS1 | NDRG1 | CHRAC1 | FGR | GPR137B | SPC25 | TGFB3 | CASP2 | APOA2 |
| PNPLA3 | EPCAM | MAN2B1 | TBC1D16 | STK24 | PPP1R1B | LRIG3 | FGB | HNRNPA3 | CENPH | TEAD2 | DZIP1L |
| TPD52 | SLC2A3 | LSP1 | ECM2 | LYRM5 | PDZD8 | VPS72 | HKR1 | ARAP3 | NDRG4 | RGS2 | CEP89 |
| TUSC1 | MMP24 | BASP1 | MAPK11 | INF2 | BCL9L | SAP30 | SH2D2A | FOXO6 | SEPP1 | SAPCD2 | CYB5A |
| NPM3 | MS4A6A | TOX | KLC4 | GORAB | SEMA4C | DUSP5 | RGS4 | HADH | TCEAL3 | REEP4 | KLC2 |
| MBNL3 | DTD1 | OIP5 | HIST1H2AD | POLR2B | KDM5B | DYNLL1 | MIS18A | SALL1 | AMT | PLEKHH2 | ITIH5 |
| ARHGEF3 | SYNGR1 | LYN | DYNC1LI2 | HNRNPH1 | ARL6IP5 | HACL1 | CEACAM6 | AREG | OLFM4 | C2orf27A | COL9A2 |
| C6orf223 | ACMSD | NIPSNAP1 | THPO | UROC1 | HCK | B4GALT2 | S100A9 | COL1A2 | LEFTY1 | UNC13D | C2orf81 |
| ATP6V0E2 | ACHE | ANXA6 | CDC25A | FZD8 | ZYX | MEGF8 | DPYSL2 | UXS1 | DPM2 | TMEM158 | PCNXL2 |
| DHCR7 | ASPM | JUNB | ANTXR1 | LAPTM4B | LRRC20 | FMOD | LBH | BTG1 | SPINT2 | COLGALT1 | CDK16 |
| CDKN2C | ST14 | OIT3 | HMHA1 | NFYA | PRCC | FXYD3 | LAMC1 | ASUN | PKHD1 | EVA1A | ITGB4 |
| TPM1 | BAG3 | DECR1 | LY86 | PTGFR | HSPB8 | NLGN2 | CADPS2 | PREPL | HNRNPA0 | C4BPB | RUNDC1 |
| DPP7 | GEM | ACSS2 | KLB | FCGRT | TRPM4 | PTPRS | LAIR1 | MAPKAPK2 | AMIGO2 | MED15 | C6orf141 |
| IDNK | SLC16A13 | GLA | LDOC1 | SCGB2A1 | ARF4 | MED14 | TUSC3 | SNX27 | CD24 | FHOD3 | VANGL1 |
| SYT7 | C1orf168 | EFNA3 | MAP1LC3B | SPRY4 | SDC1 | RGL1 | MGAT4A | PFKM | HMGXB4 | SLC7A7 | PLA2G12B |
| MYH4 | NDN | RPS19 | RRAGD | VSNL1 | LOXL4 | BATF | SRPX | IGF2BP2 | SIAH2 | PDZK1IP1 | FGFR3 |
| MFSD2A | SDS | PDK4 | HAO2 | CLIC2 | DUSP9 | ACP5 | GLDC | MUC20 | ZNF83 | C1QB | CPD |
| AVPR1A | EMILIN1 | BGN | KRT18 | TMEM150C | CPLX2 | JUN | PCP4L1 | ADAMTS2 | AGT | FBL | ANXA10 |
| CCL5 | SLC16A1 | SFN | SRGN | PDGFRA | IGLL5 | C8orf4 | RHBG | COL6A1 | MUC13 | WLS | SASH3 |
| G0S2 | AEBP1 | RAMP3 | OXCT1 | FSTL3 | UGT2B11 | C9 | ID1 | PCSK1N | SMARCC2 | PDCD1 | TSTA3 |
| CYP2C18 | SLC46A3 | HLA-DQA1 | SLC22A10 | SLC16A11 | PRG4 | GSPT2 | FAM171A1 | KLF9 | KCNS3 | VASN | TRPM8 |
| S100P | SLC25A25 | HEYL | UGT1A3 | NDRG2 | TMEM45B | GPR126 | IRF7 | QPCT | SLC9A3R2 | EIF4EBP3 | SLC28A1 |
| KRT23 | CXCL14 | SGCB | RAMP1 | PDGFA | C2orf54 | ANO1 | APCS | CDKN1C | AURKA | NPNT | AKR7A3 |
| REG1A | PAGE4 | CPVL | GBP7 | SH3RF3 | IGJ | H2AFY2 | CYP1A1 | SNCG | CYP2S1 | SCD | FIBIN |
| GDF15 | HMOX1 | TRPV4 | LRG1 | HGFAC | AKR1C1 | C1QA | PODXL | ETV4 | SOD3 | SPNS2 | PAEP |
| SAA2 | OASL | UBXN10 | PTPRF | AMACR | SPINK1 | FOS | BAIAP2L1 | AQP1 | RCN1 | LAGE3 | HLA-DOA |
| ARSE | CYP39A1 | NOTCH3 | CASC10 | ATP2A3 | CRP | IFITM2 | STMN3 | C1QC | SYT8 | PSMB8 | GJA1 |
| EFEMP1 | FASN | CYP3A7 | KLF4 | RPS8 | MME | CP | PSAT1 | KRT7 | FST | DDAH2 | CBR1 |
| BBOX1 | HLA-DRB1 | RPL39L | HSPB1 | HCLS1 | LYZ | PTGDS | CHRNA4 | TDGF1 | PLAT | SPARCL1 | PTN |
| CLDN2 | FGFR2 | HLA-DPA1 | SOWAHA | MEST | BCL2L10 | NRCAM | ATP2B2 | SRD5A1 | TCIRG1 | FGGY | C9orf16 |
| NQO1 | NEU4 | CD74 | DHCR24 | LY6E | ALDH3A1 | ME1 | RPL36A | LTBP4 | S100A14 | PSMB9 | MAFK |
| CTSF | DUSP2 | PTGR1 | CYP2A13 | THBS2 | HIBADH | SLCO1B3 | SEPT4 | PPP2R1B | GFRA1 | SULT1E1 | S100A16 |
| GMPR | VWCE | PROL1 | HOGA1 | FXYD1 | GNAZ | MDK | LUM | S100A10 | NLRP6 | C1R | STMN2 |
| HMGB3 | HLA-DQB1 | RARRES1 | HEPACAM | MAT2A | MYL9 | ENO3 | RAB17 | SPARC | SPSB1 | TJP3 | EGFL7 |
| PPP1R1A | SERPINE1 | GHR | PALLD | TMEM141 | CLEC3B | CYP1B1 | SEZ6L2 | DHRS2 | ITPKA | CD7 | ENC1 |
| SLC7A2 | MUC5B | DPP4 | SLC45A4 | SLC22A11 | CKB | PNMA6A | TNS1 | ATF5 | MAPK8IP2 | EXOC3L4 | CACNA1H |
| LGR5 | BMF | C1orf186 | FNDC5 | RNASE1 | FLNC | CRIM1 | ABHD3 | TM4SF20 | SLC17A3 | HLA-A | PLEK |
| HSPG2 | VWF | F2R | SCARB1 | THBS1 | NT5E | PID1 | CDKN3 | GCK | EBF4 | AMN | SNX10 |
| IL22RA1 | PLOD2 | HLA-DRA | FLYWCH2 | ACSM1 | LCP1 | IGFBP5 | CPNE1 | NR0B2 | PODXL2 | ZNF703 | PDE9A |
| AQP8 | LINGO1 | PERP | NAT2 | NKD1 | CYP11A1 | TCP10L | PROS1 | TKT | TFF3 | ABRACL | SULT1A2 |
| PDX1 | UPP2 | C5 | LGALS14 | SLPI | GADD45G | KRT20 | AASS | PLEK2 | HAL | MAGED1 | SLC25A18 |
| LYNX1 | UGT2B15 | CNN2 | NPDC1 | TFPI | MYOM1 | GADD45A | LTBP3 | UNC93A | AKR1C2 | LIPG | MPC1 |
| LAMB3 | KCNAB2 | BTG2 | FBXO32 | XPNPEP2 | THBS4 | PRMT6 | METTL7B | EPO | MOGAT3 | GREM2 | HLA-DMA |
| ASPN | CYP1A2 | AGPAT2 | REN | CCL25 | IFITM10 | DKK4 | CD5L | OAS3 | KRTCAP3 | ENHO | OXER1 |
| SAA1 | NECAB2 | SDCBP2 | GYG2 | CFI | RHOB | GPR37 | VNN2 | IFIT1 | EVI2B | ID3 | TPST1 |
| CD3D | PRSS8 | SLC51B | MGP | GLS2 | C2orf82 | SMO | C16orf45 | GNAS | TMEM150B | SNRPN | ABCC9 |
| HSD17B14 | GPX7 | EPB41L4B | CADM4 | MT1A | METRN | GCKR | STAT1 | NGFRAP1 | MGLL | LTBP2 | EPB41L1 |
| ANGPTL4 | ICA1 | KRT8 | HIST1H4H | UBD | SDPR | FABP4 | ATP2B4 | SOAT2 | FGF21 | LGALS2 | PDZK1 |
| ASPG | HSPA1B | PRR15L | PABPC1L | LBP | KNDC1 | INHA | SPAG5 | GGT1 | SSTR1 | MT-ND6 | SCAMP5 |
| SLC22A12 | CYP17A1 | SLC13A3 | GLUL | TCEAL8 | CYR61 | ST3GAL1 | CYP3A5 | HLA-B | MIF | ARL4A | OGDHL |
| CCL20 | IRF6 | SSTR5 | FSTL1 | TDO2 | APCDD1 | PIK3IP1 | VAT1 | GPC3 | SULT1A1 | LINC01124 | TMEM261 |
| HLA-DPB1 |  |  |  |  |  |  |  |  |  |  |  |

**Supplementary Table 4 HCC hub genes**

| BUB1 | RFC4 | NUF2 | BARD1 | YWHAQ | ZWINT | CENPA | EZH2 | CLSPN | MCM6 | DBF4 | HDAC2 |
| --- | --- | --- | --- | --- | --- | --- | --- | --- | --- | --- | --- |
| CDK1 | MAD2L1 | CDCA5 | CENPE | CDCA8 | MCM8 | NEK2 | EFTUD2 | HNRNPM | NDC80 | SPDL1 | DYNC1H1 |
| CKAP5 | PLK1 | CHEK1 | CSNK1D | CDK4 | RAN | CENPL | FOXM1 | MYBL2 | ORC1 | KIF2A | NRAS |
| TBL1XR1 | YWHAB | RACGAP1 | KIF2C | CENPO | CCNB1 | KIF18A | FBXO5 | POLA1 | ORC6 | MCM2 |  |

**Supplementary Table 5 previous ferroptosis signatures**

| Signature | Gene list | AUC (TGCA) | Reference (PMID) |
| --- | --- | --- | --- |
| 1 | AURKA, CDCA3, STMN1, SLC7A11, G6PD, NT5DC2, NQO1 | 0.751 (1-year)  0.747 (2-year)  0.744 (3-year) | 36290827 |
| 2 | AKR1C3, ATIC, G6PD, GMPS, GNPDA1, IMPDH1, PRIM1, RRM2, TXNRD1 | 0.765 (1-year)  0.684 (3-year)  0.642 (5-year) | 34377010 |
| 3 | SLC7A11, SLC1A5, TFRC, CARS1, RPL8 | 0.765 (1-year)  0.668 (3-year)  0.676 (5-year) | 35847985 |
| 4 | SLC2A1, SRXN1, FLT3, UBC, RRM2, HILPDA, KEAP1, ACSL3, PRDX6, ZFP69B, AKR1C3 | 0.800 (1-year)  0.757 (3-year)  0.746 (4-year) | 36647389 |
| 5 | HRAS, SLC2A1, NRAS, MAPK3, RRM2 | 0.758 (1-year)  0.698 (2-year)  0.658 (3-year) | 35938001 |
| 6 | G6PD, NRAS, HRAS, TIMM9, MYCN | 0.819 (1-year)  0.710 (2-year)  0.691 (3-year) | 37143953 |
| 7 | G6PD, KIF20A, EZH2, NT5DC2, SLC7A11 | 0.799 (1-year)  0.799 (2-year)  0.701 (3-year) | 40465746 |
| 8 | G6PD, SLC7A11, MYCN, KIF20A | 0.809 (1-year)  0.722 (2-year)  0.713 (3-year) | 40136455 |
| 9 | VEGFA, FANCD2, ZFP69B, EIF2S1, SLC7A11, SRXN1 | 0.76 (1-year)  0.68 (3-year)  0.66 (5-year) | 40423834 |

**Supplementary Figures and Figure Legends**

**Supplementary figure 1. Identification of the FRGs in HCC and WGCNA analysis.** (A) The DEGs between HCC and normal samples in TCGA dataset. (B) The top 20 DEGs. (C-D) Consensus clustering. (E) The clinical outcome of cluster A and cluster B HCC groups. (F) PCA analysis result. (G-H) WGCNA analysis. (I) A multifactor regulatory network based on 47 HCC-hub genes. (J-M) The impact of NRAS, CDCA8, RAN, and KIF2C level on survival of HCC patients in TCGA dataset. (N) The union of DEGs from GSE186191, GSE273819, and GSE252752. Abbreviation: exp, expression; LRAGS, lenvatinib resistance associated genes set.

**Supplementary figure 2. Validation of the FLRPS.** For ICGC HCC patients in high- and low-risk groups, we peformed the analyses of the risk score distribution and survival status (A); the level of RAN, CDCA8, KIF2C, NRAS, CENPA, YWHAB, HDAC2, and DYNC1H1 (B); the Kaplan-Meier (K-M) survival analysis (C); and the ROC analysis (D). For GSE14520 HCC patients in high- and low-risk groups, we peformed the K-M survival analysis (E) and the ROC analysis (F). (G) The impact of the FLRPS on clinical outcomes of TCGA HCC patients with different clinicopathological features, including diagnosis age, clinical stage, clinical T stage, pathological grading, and sex. (H-I) The associations between the FLRPS score and clinicopathological features, such as pathological grading and clinical stage. (J) FLRPS’ predictive performance in TCGA cancers, including esophageal, gastric, colon, and rectal adenocarcinomas. (**P < 0.05; **P < 0.01; ****P < 0.0001*). Abbreviation: OS, overall survival; AUC, area under the curve; ROC, receiver operating characteristic.

**Supplementary figure 3. The prognostic impact and mRNA expression of CENPA, RAN, KIF2C, HDAC2, DYNC1H1, NRAS and CDCA8 in HCC.** (A) The prognostic impact of CENPA, RAN, KIF2C, HDAC2, DYNC1H1, NRAS and CDCA8 in HCC patients in the Single-cell and Spatially-resolved CAncer Resources (SCAR) dataset. (B) The mRNA expression of these 8 genes in TCGA HCC tissues compared to normal tissues. (****P < 0.001*). Abbreviation: OS, overall survival.

**Supplementary figure 4. The association between FLRPS scores and the tumor immune microenvironment.** (A) FLRPS scores in different immune subtype of TCGA HCC patients. (B) The associations between FLRPS scores and mutLoad_nonsilent, CNA fraction altered, neoantigen load and mRNAsi. (C) The somatic mutation differences between high- and low-risk groups. (D-E) The landscape and frequency of copy number variations across the risk-groups. (****P < 0.001; ****P < 0.0001*). Abbreviation: mRNAsi, mRNAsi-based stemness index; CNV, copy number variations; OS, overall survival.

**Supplementary figure 5. FLRPS-linked signaling pathways.** (A-B) The gene set enrichment analysis (GSEA) of DEGs from the risk-stratified groups in TCGA and GSE14520 HCC cohorts. (C) The impact of FLRPS on clinical outcome in GSE76427-HCC cohort. (D) The GSEA of DEGs from the risk-stratified groups in GSE76427 HCC cohorts. (**P < 0.05; **P < 0.01; ***P < 0.001; ****P < 0.0001*). Abbreviation: P.val, P value.

**Supplementary figure 6. The correlation of the FLRPS and therapies.** (A) The correlation between FLRPS scores and immunotherapy outcomes. (B-C) The correlation between FLRPS scores and multi-target tyrosine kinase inhibitor (mTKI) therapy respones. (D) ROC analysis. (E) The correlation between FLRPS scores and efficacies of doxorubicin, and cisplatin. Abbreviation: AUC, area under the curve.

**Supplementary figure 7. IHC for CDCA8, DYNC1H1, CENPA, HDAC2, KIF2C, NRAS, RAN, and YWHAB in HCC patients’ tumor tissues with or without lenvatinib resistance.**

**Supplementary figure 8. YWHAB promoted lenvatinib resistances in HCC.** (A) IHC for CDCA8, DYNC1H1, CENPA, HDAC2, KIF2C, NRAS, RAN, and YWHAB in HCC patients’ tumor tissues with or without lenvatinib resistance. (B) DEGs (Top 50) between sh-YWHAB and sh-YWHAB ctrl Hepa1-6 cells. Abbreviation: sh-YWHAB, shRNA-YWHAB; sh-YWHAB ctrl, shRNA-YWHAB control.
